# Targeting Microbe-Mediated Macrophage Education: A Novel Paradigm in Cancer Immunotherapy

**DOI:** 10.34133/bmr.0294

**Published:** 2025-12-04

**Authors:** Rongwei Xu, Xinyuan Zhao, Xu Chen, Huixi Zhou, Li Cui

**Affiliations:** ^1^ Stomatological Hospital, School of Stomatology, Southern Medical University, Guangzhou 510280, Guangdong, China.

## Abstract

The tumor microenvironment (TME) is a complex ecosystem where interactions between tumor cells, immune cells, and microbes notably influence cancer progression and response to therapy. Tumor-associated macrophages (TAMs), which are crucial components of the TME, exhibit remarkable plasticity, adapting their functions in response to signals from both the tumor and its microbiota. Microbes—including bacteria, viruses, fungi, and their metabolites—modulate multiple aspects of TAM biology, from polarization and metabolism to immune modulation, thereby influencing tumor progression and immune evasion. This review focuses on the mechanisms through which microbes shape TAM responses, particularly in the context of cancer immunotherapy. Emerging therapeutic strategies leverage these microbe–TAM interactions using engineered microbes, oncolytic viruses, and microbial nanomaterials to reprogram TAMs and enhance antitumor immunity. Although formidable challenges remain, including spatial and temporal heterogeneity, mechanistic complexity, and safety concerns, these innovative approaches hold the potential to revolutionize cancer treatment. By targeting the microbe–TAM axis, this therapeutic strategy offers a promising avenue for overcoming resistance and improving the effectiveness of cancer immunotherapy.

## Introduction

The tumor microenvironment (TME) is a highly dynamic and multifaceted ecosystem that profoundly influences tumor initiation, progression, and therapeutic outcomes. Its complexity arises from the coordinated interplay between malignant cells, diverse immune populations, and stromal elements such as cancer-associated fibroblasts and endothelial cells, together with noncellular components including the extracellular matrix, soluble mediators, metabolic by-products, and the local microbiota [1]. Rather than serving as a passive scaffold, the TME actively dictates tumor behavior by regulating nutrient availability, immune surveillance, angiogenesis, and drug responsiveness [2–5]. Among its constituents, macrophages and microbes have emerged as particularly influential regulators: Macrophages exhibit remarkable plasticity and are reprogrammed into tumor-associated macrophages (TAMs) that shape immune evasion and tissue remodeling, while microbes modulate immune tone, genetic stability, and therapeutic sensitivity [6]. These interconnected influences highlight the TME not as a static background but as a dynamic and adaptive network in which macrophages and microbes represent key nodes driving tumor biology and offering promising avenues for therapeutic intervention.

Macrophages recruited into the TME frequently differentiate into TAMs, whose phenotypic states and functions exert profound influence on tumor progression and responsiveness to immunotherapy. TAMs are often skewed toward an immunosuppressive M2-like phenotype that fosters tumor growth, angiogenesis, and immune evasion. Importantly, therapeutic strategies can reprogram TAMs to shift from a protumor M2 phenotype toward an antitumor M1 phenotype, thereby restoring antitumor immunity. For instance, inhibition of colony-stimulating factor-1 receptor reduces TAM infiltration and alleviates immunosuppressive pressure, whereas stimulation with Toll-like receptor (TLR) agonists or interferon-γ (IFN-γ) activates TAMs to produce proinflammatory cytokines, enhancing T cell-mediated cytotoxicity [7,8]. Beyond signaling cues, targeting TAM metabolism provides an additional layer of intervention; inhibition of arginase-1 (ARG1) or induction of inducible nitric oxide synthase (iNOS) activity effectively reshapes TAM function toward an inflammatory, tumoricidal state [9,10]. More recently, accumulating evidence indicates that the tumor-associated microbiota can indirectly modulate antitumor immunity by influencing TAM activity and polarization, revealing new opportunities to exploit microbe–macrophage crosstalk as a therapeutic axis within the TME.

Microbes within the TME, including bacteria, fungi, and viruses, have emerged as critical determinants of tumor biology and immune regulation. Distinct microbial communities are now recognized across diverse malignancies such as colorectal, lung, and breast cancers, with their composition and functional impact varying according to tumor type and microenvironmental context. *Fusobacterium nucleatum*, a gut commensal enriched in colorectal cancer (CRC), exemplifies this by promoting tumor growth and suppressing antitumor immunity [11]. In lung cancer, *Streptococcus pneumoniae* has been implicated in shaping tumor progression through the modulation of inflammatory responses [12]. Certain microbes exert direct oncogenic effects: Human papillomavirus (HPV) integrates its genome into host DNA, expressing E6 and E7 oncoproteins that subvert cell cycle checkpoints and drive cervical carcinogenesis [13]. Beyond their direct influence on tumor cells, microbes also orchestrate immune regulation within the TME. *Bifidobacterium* promotes dendritic cell (DC) maturation and antigen presentation, thereby enhancing T cell activation and effector differentiation [14]. In contrast, *F. nucleatum* activates the nuclear factor κB (NF-κB) pathway, leading to the up-regulation of CXCL2. This CXCL2-mediated crosstalk promotes the polarization of TAMs toward an immunosuppressive M2 phenotype, which dampens antitumor immunity [15]. Collectively, these findings highlight the dualistic nature of the tumor microbiome, with both immunostimulatory and immunosuppressive effects, and underscore the emerging recognition that microbe–TAM crosstalk constitutes a promising therapeutic axis in cancer treatment.

In this review, we examine the intricate dynamics of the TME, with particular emphasis on how microbial communities influence the phenotypic and functional states of TAMs. We first delineate the diverse roles of TAMs in tumor progression and immune regulation, and then consider the remarkable heterogeneity of microbes within the TME, highlighting how their interactions with immune and stromal components shape tumor biology. Importantly, we underscore emerging strategies that specifically target the microbe–TAM axis as a novel therapeutic frontier. These include the use of engineered microbes to remodel immune tone, microbial-derived nanomaterials to deliver immunomodulatory cues, and oncolytic viruses (OVs) to simultaneously debulk tumors and reprogram the immune landscape. By integrating these advances, this review aims to illuminate an underexplored but increasingly compelling paradigm: leveraging the interplay between microbes and TAMs as a means of expanding the scope of cancer immunotherapy. Such approaches move beyond conventional tumor- or immune cell-centric interventions, offering new conceptual and technological avenues to enhance treatment efficacy and overcome resistance.

## Macrophages in the TME

### Origin and heterogeneity of macrophages

Macrophages are indispensable components of the innate immune system and are present in virtually all tissues, where they form highly specialized subpopulations defined by their anatomical location and functional programs. They arise from 2 principal ontogenies: embryonic erythro-myeloid progenitors in the yolk sac and fetal liver, and bone marrow-derived monocytes that emerge in adult life. Tissue-resident macrophages, such as microglia in the brain, Kupffer cells in the liver, and Langerhans cells in the skin, largely originate from embryonic precursors and are maintained through self-renewal independently of adult hematopoiesis. These long-lived macrophage populations are central to tissue development, homeostasis, and immune regulation [16]. By contrast, circulating monocytes can be rapidly mobilized in response to chemokine signals such as CCL2, infiltrate tissues, and differentiate into macrophages that contribute to host defense, repair, and inflammation [17,18].

Within the TME, macrophage biology becomes even more complex. TAMs are predominantly replenished by recruited monocytes and display remarkable phenotypic and functional diversity, ranging from promoting tumor growth, angiogenesis, and immune evasion to, in some contexts, exerting antitumor activity [19–21]. Distinct subsets can also be defined by disease stage, for example, metastasis-associated macrophages exhibit specialized functions that differ from TAMs in primary tumors, underscoring how tissue context and tumor evolution shape macrophage identity [22]. This extraordinary plasticity renders TAMs both indispensable participants in tumor progression and attractive therapeutic targets, as their density and polarization states strongly correlate with clinical outcomes.

Recent technological advances have greatly expanded our understanding of TAM heterogeneity. High-resolution single-cell and spatial transcriptomic analyses have revealed that human breast and colon tumors harbor at least 5 spatially segregated macrophage populations whose niches are conserved across health and disease. These include IL4I1^+^ macrophages that engulf apoptotic cells in high-turnover regions and predict favorable prognosis in colon cancer, SPP1^+^ macrophages confined to hypoxic or necrotic tumor cores and associated with poor outcome, FOLR2^+^ macrophages embedded within plasma cell niches, and NLRP3^+^ macrophages that colocalize with neutrophils to drive inflammasome activation [23]. Complementing these spatial maps, temporally resolved profiling has demonstrated that canonical circadian programs in macrophages are profoundly disrupted by tumor-derived acidification and lactate, generating heterogeneous circadian phases within the TAM pool; strikingly, restoration of circadian coherence in murine pancreatic tumors markedly restrains tumor growth [24]. Collectively, these findings highlight that macrophage ontogeny, spatial positioning, and temporal regulation together shape their profound heterogeneity in tumors. A nuanced understanding of these dimensions is essential for the rational design of therapeutic strategies aimed at reprogramming macrophage function in cancer and other disease contexts.

### Polarization states of macrophages

Building on their diverse ontogeny and tissue-specific identities, macrophages display extraordinary functional plasticity, enabling them to adopt distinct polarization states in response to environmental signals. This adaptability is most commonly described along the M1–M2 axis, a conceptual framework that, while simplified, captures key aspects of their divergent roles in immunity, homeostasis, and disease [25].

Classically activated M1 macrophages are induced by proinflammatory cues such as IFN-γ, lipopolysaccharide (LPS), and microbial products. They are characterized by the robust production of inflammatory mediators, including tumor necrosis factor-α (TNF-α), interleukin-1β (IL-1β), IL-12, and reactive oxygen species (ROS), which collectively promote T helper 1 (Th1) cell responses and exert potent antimicrobial and antitumor activities. Mechanistically, IFN-γ engagement with its receptor activates the Janus kinase (JAK)–signal transducer and activator of transcription (STAT) pathway, driving the expression of hallmark M1-associated genes such as TNF-α and IL-6 [26]. In contrast, alternatively activated M2 macrophages arise in response to cytokines such as IL-4, IL-10, IL-13, and transforming growth factor-β (TGF-β). These cells are associated with immunoregulatory and tissue-repair functions and are further subdivided into M2a, M2b, and M2c subtypes, each serving specialized roles in wound healing, immune modulation, and pathological remodeling [27]. For instance, M2 macrophages express high levels of ARG1, thereby promoting tissue repair and fibrosis [28,29]. Within the TME, M2-polarized TAMs typically facilitate tumor progression by fostering angiogenesis, remodeling the extracellular matrix, and secreting immunosuppressive factors such as IL-10 and TGF-β, which blunt antitumor immune responses [30].

Crucially, macrophage polarization is not fixed. The dynamic plasticity of these cells allows them to transition between M1 and M2 phenotypes in response to shifts in the cytokine milieu, metabolic cues, or microbial signals. This fluidity underscores the importance of local context in shaping macrophage identity and highlights their potential as therapeutic targets. Strategies that reprogram TAMs from an M2 phenotype, protumor state to M1 phenotype, antitumor phenotype represent a promising avenue to both regulate inflammation and modulate tumor progression.

### Functional roles of macrophages in the TME

Within the TME, macrophages fulfill diverse and context-dependent functions, acting as both facilitators of malignant progression and modulators of antitumor immunity. TAMs, which constitute one of the most abundant immune cell populations in tumors, frequently acquire M2-like phenotypes that sustain tumor growth, angiogenesis, and immune evasion. These immunosuppressive macrophages secrete a repertoire of cytokines, chemokines, and growth factors that orchestrate tumor-promoting circuits and remodel the immune landscape.

Mechanistic studies have revealed how M2-like TAMs potentiate malignancy through specialized signaling programs. Single-cell analyses of gallbladder cancer demonstrated that TAM-derived CCL2 engages CCR2 on tumor cells, activating a mitogen-activated protein kinase (MAPK) kinase (MEK)–extracellular signal–regulated kinase (ERK)–ELK1–SNAIL cascade that amplifies epithelial–mesenchymal transition (EMT) and cancer stemness while simultaneously recruiting additional monocytes to reinforce an M2-enriched niche [31]. Similarly, in ovarian cancer, M2-like TAMs secrete epidermal growth factor (EGF) to suppress the metastasis-restraining long noncoding RNA (lncRNA) LIMT via EGF receptor (EGFR)–ERK signaling, thereby accelerating proliferation, migration, and EMT; importantly, pharmacologic EGFR blockade or enforced LIMT expression reverses these protumoral effects [32]. Beyond cytokine and growth factor signaling, TAMs also regulate immune surveillance through efferocytosis: neuropilin-2 (NRP2) expressed on TAMs drives the immunologically silent clearance of apoptotic tumor cells, suppressing secondary necrosis and thereby limiting infiltration of CD8^+^ T and natural killer (NK) cells. Disruption of this NRP2 axis restores antitumor immunity and curtails tumor growth [33]. Together, these studies highlight how TAMs promote malignancy through a multifaceted arsenal of chemokines, growth factors, and phagocytic regulators, underscoring the need for multimodal therapeutic strategies to dismantle their support of tumor progression.

**Fig. 1.**
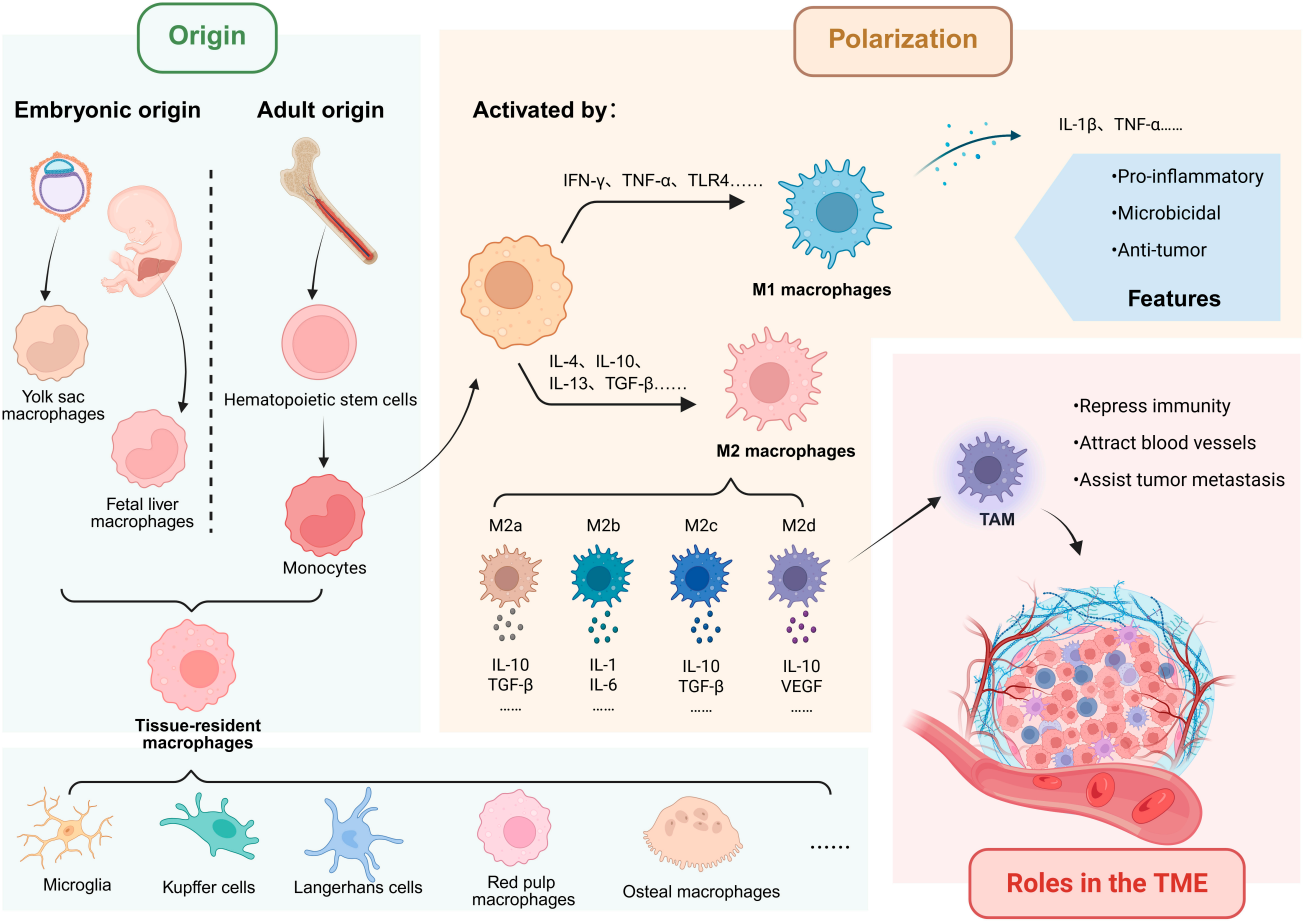
Origins, polarization, and functions of macrophages in the TME. Macrophages are derived from 2 major sources: embryonic progenitors originating from the yolk sac and fetal liver, which give rise to tissue-resident macrophages such as microglia and Kupffer cells, and monocytes derived from the bone marrow that infiltrate tissues during adulthood. Within the TME, macrophages demonstrate marked plasticity and can be polarized into distinct functional phenotypes in response to various cytokines and environmental signals. Classical activation (M1), induced by IFN-γ, TNF-α, or TLR agonists, is associated with proinflammatory, microbicidal, and antitumor immune responses. In contrast, alternative activation (M2), driven by IL-4, IL-10, IL-13, or TGF-β, contributes to immunosuppression, angiogenesis, and tumor progression. M2 macrophages can be further classified into subtypes, each with specific functional roles. TAMs within the TME promote tumor growth through multiple mechanisms, including cytokine production, efferocytosis, metabolic reprogramming, and facilitation of metastasis. Their phenotypic flexibility presents potential opportunities for therapeutic strategies aimed at reprogramming TAMs toward antitumor states.

Importantly, macrophage polarization is not static. Their dynamic plasticity allows transitions between M1 and M2 phenotype in response to metabolic and cytokine cues within the TME. For example, activation of peroxisome proliferator-activated receptor α (PPARα) enhances macrophage phagocytic and bactericidal activity while promoting CD8^+^ T cell priming. In colorectal and breast cancers, pharmacologic suppression of cancer stemness using salinomycin, SB-431542, JIB-04, or napabucasin reduces Wnt/β-catenin, TGF-β, histone demethylase, and STAT3 signaling, thereby depriving TAMs of tumor-derived instructive cues and reprogramming them from an M2 to an M1 phenotype. This phenotypic switch coincides with the loss of stemness markers CD133 and CD44, illustrating a bidirectional circuit in which cancer cells and macrophages reinforce one another’s states, and which can be therapeutically interrupted [34]. In parallel, metabolic checkpoints also govern TAM function: The fatty acid sensor GPR84 functions as a metabolic rheostat, with genetic deletion attenuating STAT1 activity and enforcing an anti-inflammatory state, whereas pharmacologic activation with the synthetic agonist 6-OAU amplifies STAT1 signaling and robustly drives M1 polarization. Treatment with 6-OAU not only suppresses tumor growth as monotherapy but also synergizes with anti-PD-1 therapy by converting the TME from immunosuppressive to immunostimulatory [35]. Collectively, these insights establish TAMs as central regulators of the TME, equipped with both tumor-promoting and immune-modulating capacities. Their functional versatility makes them highly tractable targets for therapy, and interventions that reprogram TAMs from M2 phenotype, protumoral phenotype into M1 phenotype, immunostimulatory state hold considerable promise for enhancing the efficacy of cancer immunotherapy (Fig. 1).

## Microbes in the TME

### Composition and diversity of the microbes in the TME

Distinct microbial communities have now been identified across a wide spectrum of malignancies, with each tumor type harboring a unique microbial signature, even in tissues without direct exposure to the external environment [36]. These findings challenge the traditional view of tumors as sterile sites and suggest that microbial colonization is a generalizable, though heterogeneous, feature of human cancers.

In breast cancer, bacterial DNA profiling has revealed pronounced enrichment of Proteobacteria and Actinobacteria within tumor tissues compared with adjacent nontumoral counterparts, with marked compositional shifts across tumor stages and molecular subtypes. Such distinctions underscore the potential of microbial profiles to inform disease progression and prognosis [37]. In gastrointestinal malignancies, CRC tissues display higher abundances of Firmicutes and Clostridia relative to healthy controls [38]. Notably, microbial variations extend beyond the tumor bed, with distinct signatures observed between proximal and distal lesions, reflecting spatial heterogeneity within the colon [39]. Pancreatic ductal adenocarcinoma exemplifies another striking case of microbial dysregulation, in which gut bacteria migrate through pancreatic ducts into the TME. This translocation promotes local immunosuppression, characterized by expansion of Proteobacteria and depletion of butyrate-producing commensals with antitumor potential [36]. Respiratory tumors likewise harbor distinct microbial landscapes. Lung cancer tissues are enriched in Proteobacteria [40], while the detection of *Lactobacillus acidophilus* in the lower respiratory microbiome has recently been proposed as a predictive biomarker for lung cancer risk in patients with diffuse parenchymal lung disease [41]. Taken together, these observations highlight the diagnostic promise of tumor-specific microbiome signatures, which may complement existing biomarkers in oncology.

Beyond primary tumors, emerging evidence indicates that metastatic lesions retain microbial features inherited from their tissue of origin but also undergo selection shaped by the metastatic niche. Hypoxic microenvironments, for example, preferentially sustain anaerobes such as Fusobacterium, which in turn may drive therapeutic resistance, particularly to immunotherapy [42]. These insights not only deepen our understanding of microbial diversity in the TME but also emphasize its clinical relevance, as tumor–microbe associations could influence disease course, metastatic patterns, and treatment outcomes. Although comprehensive analyses of microbiome–cancer interactions from immunological and oncological perspectives have been provided in prior reviews [43], the present focus lies in elucidating how these microbial ecosystems interface with immune regulation—especially macrophage biology—to shape tumor fate.

### Association between microbes and tumor progression

Tumor-associated microbiota contribute to oncogenesis and malignant progression through diverse and multifaceted mechanisms, ranging from the production of metabolites that reshape the TME to the direct modulation of metastatic dissemination. Increasing evidence underscores microbial metabolites as critical mediators of tumor–host interactions. For instance, *Escherichia coli* KUB-36, a non-exotoxigenic strain with established intestinal safety, exhibits selective anticancer activity through its extracellular metabolites. Its short-chain fatty acid-dominated profile, enriched in acetic acid, displays potent cytotoxicity against MCF-7 breast cancer cells while sparing normal mammary epithelial cells and exerting weaker effects on colon cancer and leukemia cells [44]. This strain-specific activity illustrates how microbial metabolic heterogeneity can be harnessed as a source of anticancer therapeutics.

In the immunomodulatory subtype of triple-negative breast cancer, the microbial metabolite trimethylamine N-oxide (TMAO), produced by Clostridiales, has been linked to enhanced antitumor immunity. Elevated TMAO levels correlate with increased CD8^+^ T cell infiltration, and mechanistic studies demonstrate that TMAO induces gasdermin E (GSDME)-mediated pyroptosis in tumor cells, releasing inflammatory cytokines that further activate tumor-specific CD8^+^ T cells. Remarkably, dietary replenishment of choline precursors elevates systemic TMAO, augments CD8^+^ T cell responses, and restrains tumor growth, highlighting a novel paradigm whereby microbial metabolites induce tumor pyroptosis to potentiate immune infiltration [45].

Beyond metabolic crosstalk, microbes also shape the metastatic trajectory of tumors, a defining hallmark of malignancy. Metastasis requires tumor cells to detach from the primary site, survive in circulation under mechanical stress and immune surveillance, and colonize distant organs. Recent evidence indicates that microbiota actively participate in this process. In MMTV-PyMT spontaneous breast cancer models, intracellular bacteria within circulating tumor cells activate RhoA–ROCK signaling to reorganize the actin cytoskeleton, conferring resilience to hemodynamic shear stress. Notably, microbiota depletion reduces lung metastases without affecting primary tumor growth, directly implicating intratumoral bacteria in metastatic dissemination [46]. Extending this, spatial multi-omics approaches have revealed niche specialization by intratumoral microbes. In human breast cancers, *F. nucleatum* preferentially colonizes tumor cell-dense regions, where GeoMx spatial profiling demonstrates enrichment of transcripts and proteins associated with proliferation, migration, and invasion. VEGFD and PAK1, in particular, display strong positive correlations with bacterial load, and integrated coculture analyses confirm that MAPK signaling, mediated by VEGFD and PAK1, is a key driver of bacteria-facilitated tumor progression and metastasis [47].

**Fig. 2.**
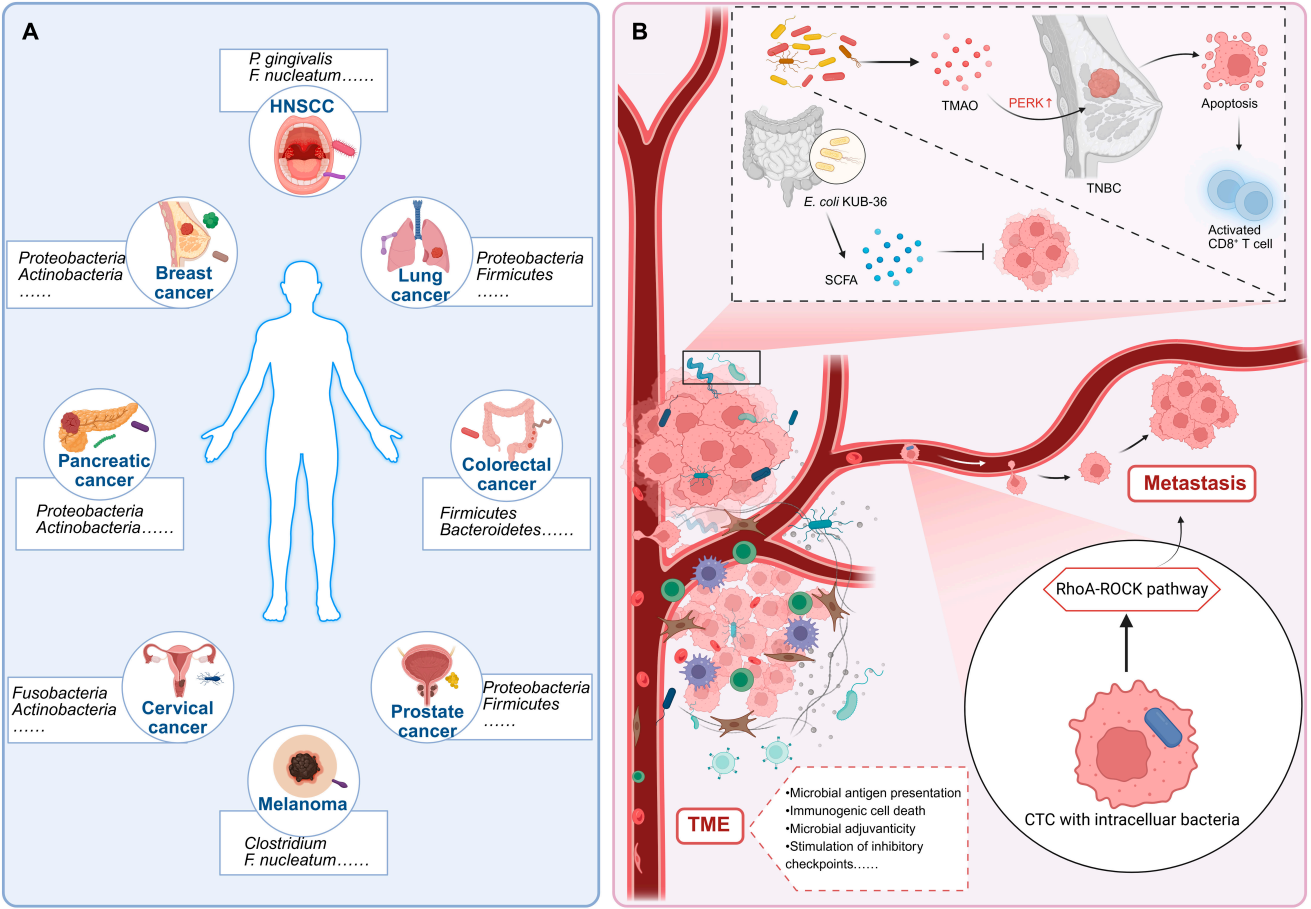
Microbiome composition across cancer types and functional roles in tumor progression. (A) Different cancer types harbor distinct and specific microbial communities, forming unique tumor type-specific microbiome signatures. (B) Intratumoral microbes and their metabolites facilitate tumor progression through diverse mechanisms including modulation of metastatic pathways, immunomodulatory metabolite production, and strain-specific cytotoxic effects. Key processes influenced include cytoskeletal reorganization promoting metastasis, pyroptosis-mediated T cell activation, and direct cytotoxicity against tumor cells. Furthermore, microbes impact antitumor immunity through antigen presentation, ICD, and checkpoint stimulation. Collectively, these multifaceted interactions highlight the crucial role of the tumor microbiome in shaping cancer progression and the immune microenvironment.

The immunomodulatory roles of microbes in cancer are equally indispensable. Previous systematic studies have delineated diverse mechanisms, including microbial antigen presentation, cross-reactivity with tumor antigens, induction of immunogenic cell death (ICD), adjuvant-like activity via pattern recognition receptors, production of immunoregulatory metabolites, and activation of inhibitory checkpoints [48]. Given the breadth of this existing literature, these mechanisms will not be further elaborated here. Instead, the focus of this review shifts to the intersection of microbes and macrophages. As macrophages represent a central hub in orchestrating immune responses within the TME, understanding how microorganisms modulate their function provides a critical lens through which to explore novel therapeutic opportunities (Fig. 2).

## Influence of Tumor Microbes on Macrophage Behavior

### Effects of bacteria on macrophage behavior

#### Modulation of TAM secretion

Bacterial-derived cues exert profound and multifaceted influences on macrophage function, encompassing the modulation of cytokine secretion, the regulation of proliferative and chemotactic responses, and the reprogramming of polarization states. Among these, the ability of microbes to reconfigure macrophage secretory programs represents a particularly critical mechanism by which they shape immune balance, inflammation, and tumor progression.

Macrophages are inherently versatile secretory cells, producing a broad array of cytokines and soluble mediators that orchestrate immune regulation, tissue repair, and host defense. Specific commensal and pathogenic bacteria can rewire this secretory activity with striking consequences for disease outcomes. In inflammatory bowel disease, for example, an adherent *E. coli* strain was shown to enhance IL-10 secretion by macrophages, establishing an anti-inflammatory circuit that reduced intestinal inflammation and suppressed tumorigenesis. This protective effect required both macrophage-derived IL-10 and downstream signaling to intestinal epithelial cells, highlighting the therapeutic potential of selectively inducing anti-inflammatory macrophage secretion in chronic inflammation and cancer [49].

Conversely, microbial signals can also hijack macrophage secretory pathways to fuel tumor-promoting inflammation. In colitis-associated cancer, gut microbiota-derived LPS reprogrammed macrophage cytokine output through a 2-step process: first stimulating colonic epithelial cells to release CCL2, which recruited monocyte-like macrophages (MLMs) to the tumor site, and subsequently activating these MLMs to secrete high levels of IL-1β. This macrophage-derived IL-1β amplified IL-17 production by T helper cells, fostering a protumorigenic inflammatory milieu [50]. In head and neck squamous cell carcinoma (HNSCC), pathogen-derived signals illustrate yet another facet of bacterial control over macrophage secretory behavior. Exposure to *Porphyromonas gingivalis* LPS selectively augmented macrophage nitric oxide (NO) secretion while suppressing TNF-α release. The conditioned medium from these reprogrammed macrophages inhibited proliferation in one primary tumor line (HN4) but consistently enhanced invasion across all tested primary and metastatic HNSCC lines. These findings reveal how oral pathogen-derived molecules can reconfigure macrophage secretory output to promote tumor aggressiveness, particularly invasive behavior [51]. Together, these studies illustrate the dualistic capacity of bacterial-derived cues to either constrain or exacerbate disease by sculpting macrophage secretion profiles. They also underscore the potential of therapeutically targeting these microbe–macrophage interactions to restrain protumorigenic inflammation while preserving or enhancing protective immune functions.

#### Impact on TAM proliferation and migration

In addition to modulating macrophage secretory functions, bacteria play an essential role in regulating macrophage behaviors critical for tumor progression, including proliferation, activation, and migration. This phenomenon is particularly evident in the context of *F. nucleatum* subspecies *animalis* (Fna), a pathogen frequently associated with human CRC. Fna induces the expression of CCL20 in both cancer cells and monocytes, promoting the recruitment and activation of monocyte/macrophages. This pathogen-driven macrophage activation is further potentiated by elevated levels of proinflammatory cytokines such as IL-17A and TNF-α, which together foster a tumor-promoting microenvironment [52]. These findings illustrate how bacterial infections can reprogram fundamental macrophage biology, actively driving colorectal carcinogenesis.

Similarly, distinct macrophage profiles in ovarian cancer subtypes correlate with tumor prognosis and microbial composition. Immune-enriched ovarian cancer, which is characterized by an abundance of M1 macrophages and associated with a better prognosis, harbors a unique intratumoral microbiota. A striking correlation was found between M1 macrophage levels and the presence of specific bacterial species, including *Achromobacter deleyi*, *Microcella alkaliphila*, *Devosia sp.* strain LEGU1, *Ancylobacter pratisalsi*, and *Acinetobacter seifertii*. Functional validation experiments revealed that *A. seifertii* directly inhibits macrophage migration, underscoring how tumor-resident microbes influence macrophage spatial distribution and subsequently impact antitumor immunity. These microbial signatures not only are associated with macrophage activity but also serve as predictors of ovarian cancer prognosis [53]. Collectively, these studies demonstrate that microbes residing in the TME can orchestrate key macrophage functions, such as migration and proliferation, thereby shaping the immune landscape and influencing tumor progression. The ability of bacteria to modulate macrophage behavior further highlights their critical role in tumor biology, offering new therapeutic avenues for targeting microbe–macrophage interactions to control tumor growth and metastasis.

#### Influence on TAM phenotypic polarization

Bacteria and their metabolites play a pivotal role in reprogramming macrophage polarization, facilitating the transition from immunosuppressive M2 to immunostimulatory M1 phenotypes, which can enhance antitumor immunity. For example, *Lactiplantibacillus plantarum* IMB19, a gut-derived bacterium, activates TLR2 signaling in TAMs via its capsular polysaccharide. This stimulation drives M1 polarization, resulting in the sequestration of iron through the iron transporter lipocalin-2, which in turn amplifies CD8^+^ T cell responses, promoting a more robust antitumor immune environment [54]. Similarly, another bacterial component, FimH—an adhesin from *E. coli* —has been shown to promote M1 macrophage activation and repolarize M2 macrophages toward M1 phenotype via the TLR4/MD2 pathway. This reprogramming enhances inflammatory responses within the TME and synergizes with anti-PD-L1 therapy to bolster antitumor immunity [55]. In a parallel manner, *Akkermansia muciniphila*—a gut commensal bacterium—also demonstrates potent immunomodulatory effects through macrophage reprogramming. It activates TLR2 signaling, leading to NF-κB-mediated NLRP3 inflammasome activation and subsequent polarization of M1 phenotype macrophages. This mechanism suppresses colorectal tumorigenesis in murine models, and its clinical relevance is supported by positive correlations between *A. muciniphila* abundance, NLRP3/TLR2 expression, and M1 macrophage infiltration in CRC patients [56]. Further extending this paradigm, the gut microbiota-derived metabolite TMAO has been shown to directly reprogram macrophages by activating the type I interferon pathway. This reprogramming boosts the production of proinflammatory cytokines, such as IL-6 and IL-12p40, while concurrently suppressing M2 markers, including Arg1, Fizz1, and Mgl1. This shift in macrophage polarization synergizes with immune checkpoint blockade, providing a potential therapeutic avenue for enhancing cancer immunotherapy [57].

**Fig. 3.**
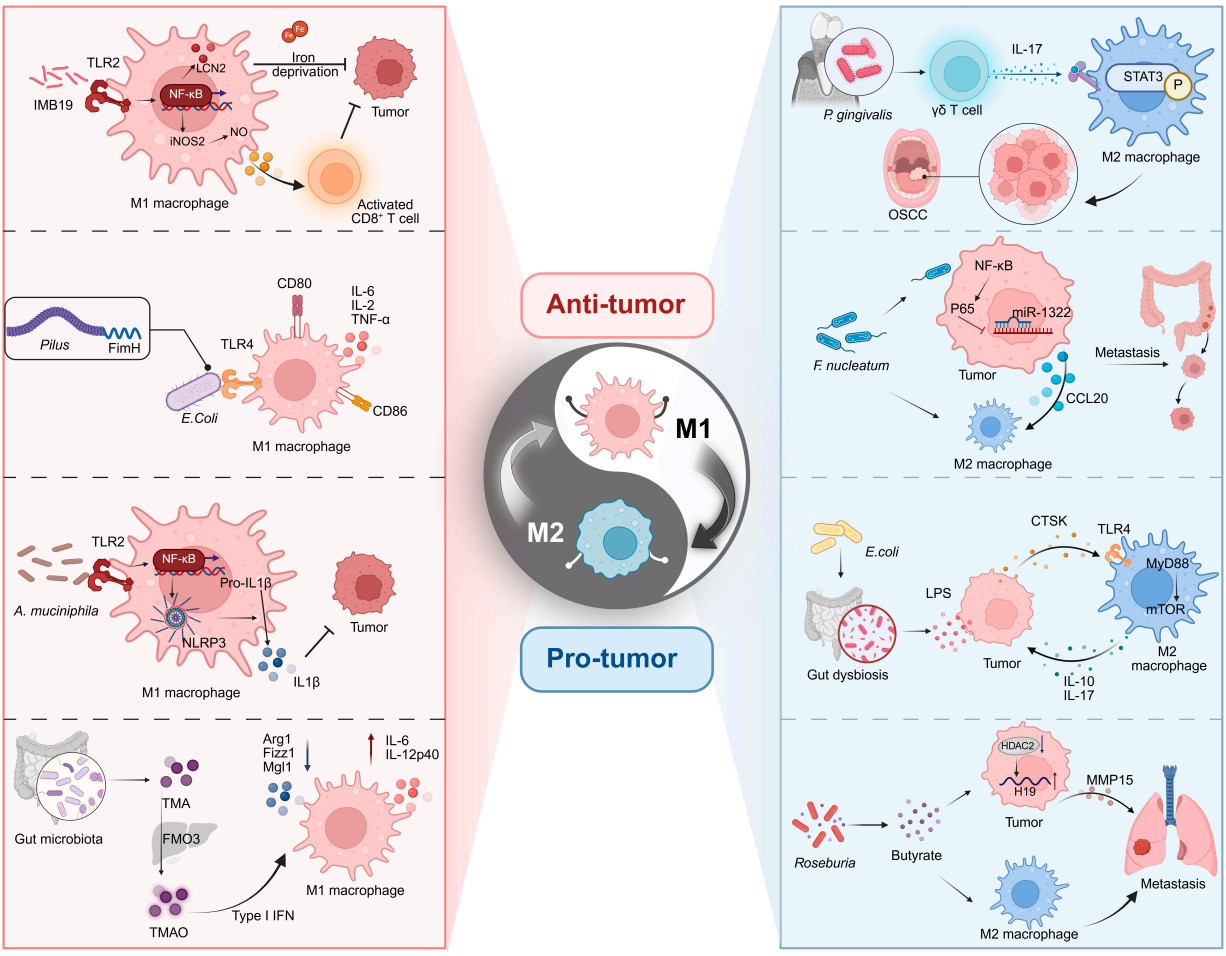
Bidirectional regulation of TAM polarization by the tumor microbiome. The intratumoral microbiota exert opposing effects on macrophage polarization through distinct molecular pathways. Microbial components and metabolites—including capsular polysaccharide of *Lactiplantibacillus* IMB19, adhesins such as FimH, and metabolites like TMAO—promote M1 polarization via TLR2/4-dependent NF-κB activation, inflammasome formation, and type I interferon signaling. In contrast, whole pathogens including *Lactiplantibacillus* and *F. nucleatum* , along with bacteria-derived molecules such as LPS and butyrate, induce M2 polarization through IL-17–STAT3 activation, CTSK/mTOR signaling, and HDAC-mediated epigenetic modification. These reciprocal interactions highlight the microbiota’s potent role in directing immune responses within the TME and offer strategic avenues for therapeutic intervention.

Conversely, many bacterial species also drive protumorigenic M2 polarization via diverse immunosuppressive mechanisms. In oral squamous cell carcinoma (OSCC), the periodontitis-associated pathogen *P. gingivalis* activates IL-17^+^ γδ T cells, which, through STAT3 signaling, induce M2 TAM infiltration, thereby accelerating tumor growth [58]. In CRC, *F. nucleatum* promotes metastasis by down-regulating miR-1322, which in turn up-regulates CCL20, leading to the recruitment and polarization of macrophages to the M2 phenotype [59]. Additionally, gut dysbiosis, driven by *E. coli*, elevates LPS levels, triggering tumor-secreted cathepsin K (CTSK). CTSK activates M2 polarization through TLR4/mechanistic target of rapamycin (mTOR) signaling, creating a prometastatic feedback loop [60]. In lung cancer, intratumoral butyrate-producing bacteria, such as *Roseburia*, epigenetically up-regulate H19 via histone deacetylase 2 (HDAC2) inhibition, thereby driving M2 polarization and promoting recurrence [61]. Collectively, these studies underscore the profound impact of microbial cues on macrophage polarization within the TME. The ability of bacteria to induce either pro- or antitumorigenic polarization in TAMs through various signaling pathways and metabolites highlights the therapeutic potential of modulating these interactions. By recalibrating macrophage phenotypes, microbial signals can be harnessed to influence tumor progression, offering new avenues for targeted immunotherapies (Fig. 3).

#### Probiotic effects on TAM behavior

Probiotics, which are commensal microbes essential for gut–immune crosstalk, have emerged as key modulators of macrophage function through conserved signaling pathways. These bacteria can directly influence macrophage polarization and activation, leveraging their interactions with immune cells to modulate systemic inflammation and tumor immunity. One illustrative example involves *Lactococcus lactis* C60, which shifts intratumoral macrophages toward a glycolysis-dominant metabolic state via TLR sensing of bacterial components. This metabolic shift elevates adenosine triphosphate (ATP) levels, driving M1 polarization and promoting CD8^+^ T cell priming in melanoma, thereby enhancing antitumor immunity [62].

Similarly, the heat-killed strain *Levilactobacillus brevis* KU15159 activates MAPK signaling in macrophages, amplifying the secretion of proinflammatory mediators such as NO, TNF-α, IL-1β, and IL-6. This activation also enhances macrophage phagocytic activity, reinforcing the immune response in tumor models [63]. In a related study, *Lactobacillus sakei* K040706 engages the TLR2/NF-κB signaling cascade to boost macrophage effector functions, including iNOS/NO production and cytokine release, thereby restoring immune competence in immunosuppressed models [64].

Despite these promising findings, the immunostimulatory effects of probiotics on macrophage polarization and activation remain largely unvalidated within the TME, where the complex interplay between microbial cues, immune cells, and tumor cells can vary considerably. This underscores a critical frontier for future oncological research—understanding how probiotics can be leveraged in the TME to reprogram macrophage functions and enhance antitumor immunity. Investigating the translational potential of these microbes in cancer therapy will be pivotal for harnessing their full immunotherapeutic potential.

### Effects of the virus on macrophage behavior

#### Epstein–Barr virus

Epstein–Barr virus (EBV), an oncogenic herpesvirus, plays a crucial role in shaping the TME by manipulating macrophage polarization and function, thereby fostering an immunosuppressive milieu across multiple malignancies. Clinical evidence in nasopharyngeal carcinoma (NPC) underscores EBV’s substantial influence on macrophage dynamics. In EBV-positive tumors, particularly those expressing EBER1 (which accounts for 90% of cases), increased levels of macrophage migration inhibitory factor (MIF) correlate with distinct spatial segregation of macrophage subsets: M1 macrophages (CD11c^+^) are predominantly found in tumor nests, while M2 macrophages (CD163^+^) are enriched in the stroma. Furthermore, co-infection with HPV further modulates the infiltration of M1 macrophages, complicating the immune landscape of NPC [65].

Mechanistically, EBV manipulates macrophage differentiation via its viral lytic proteins. In NPC, the abortive lytic cycle—induced by the transcription activator ZTA—drives the secretion of proinflammatory mediators such as granulocyte-macrophage colony-stimulating factor (GM-CSF), IL-8, and GRO-α. These cytokines recruit monocytes and reprogram them into proangiogenic, invasive M2-like TAMs, promoting tumor growth and metastasis [66]. Additionally, EBV nuclear antigen 1 (EBNA1) plays a pivotal role in directly inducing M2 polarization in NPC. The resultant M2 macrophages not only enhance tumor progression but also convert naïve T cells into immunosuppressive T regulatory cells (Tregs), amplifying immune evasion and further reinforcing the immunosuppressive TME [67].

**Fig. 4.**
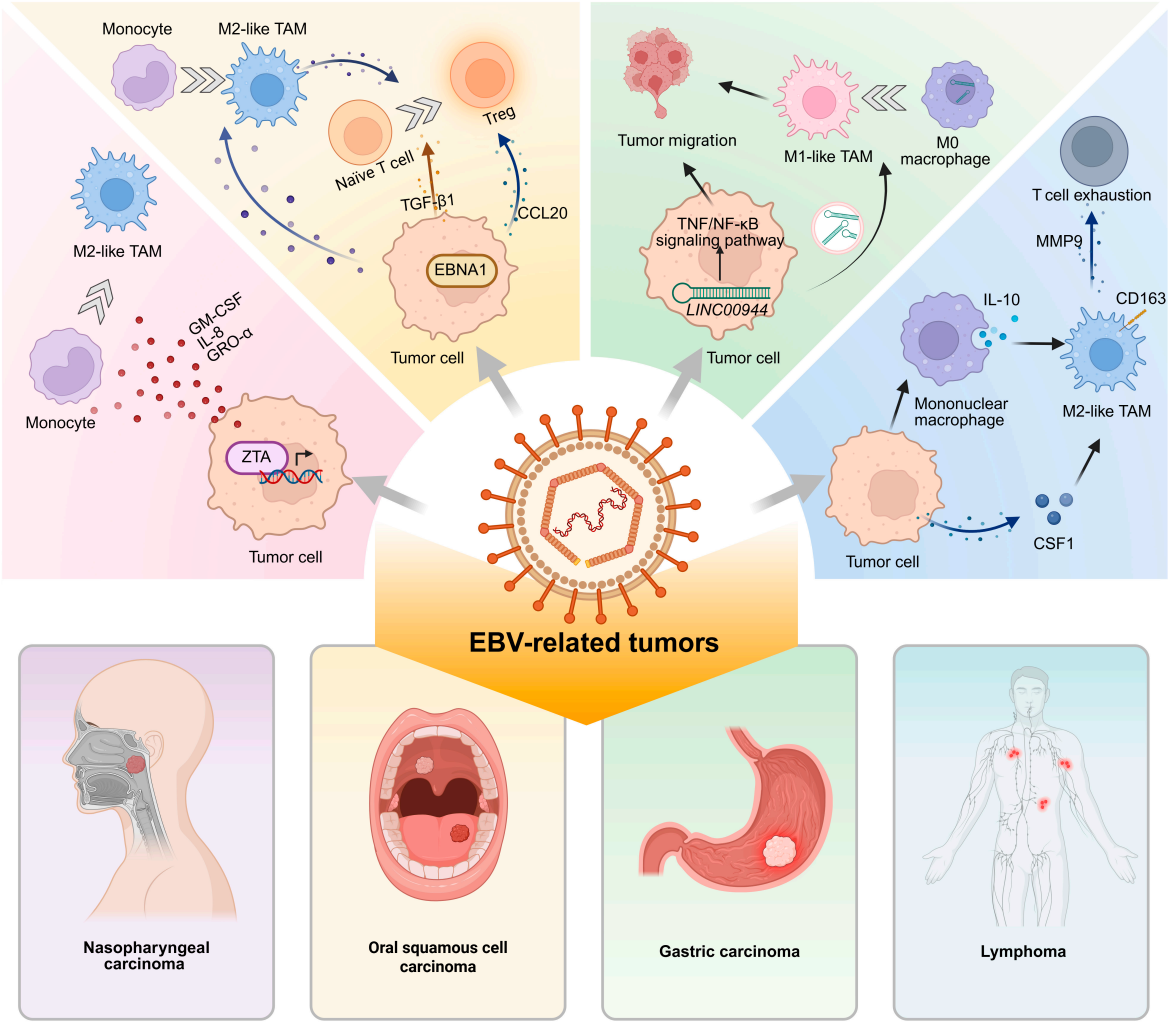
EBV-mediated reprogramming of TAMs and associated malignancies. EBV-infected tumor cells express viral proteins, such as EBNA1 and ZTA, which induce the production of host-derived factors like TGF-β1 and CCL20. These factors facilitate the differentiation of monocytes into M2-like TAMs. These TAMs display an immunosuppressive phenotype, characterized by elevated expression levels of IL-10 and CD163, thereby contributing to T cell exhaustion and compromised antitumor immunity. This reprogramming initiated by EBV represents a key feature of EBV-associated malignancies, such as nasopharyngeal carcinoma, gastric carcinoma, oral squamous cell carcinoma, and lymphoma, in which it promotes immune evasion and tumor progression.

Beyond NPC, EBV has similarly been shown to subvert macrophage function in other cancers. In OSCC, EBV-induced lncRNA LINC00944 enhances tumor invasiveness by promoting M1 macrophage differentiation through extracellular vesicle-mediated NF-κB signaling. This paradoxically amplifies local inflammation while driving tumor progression [68]. In EBV-positive primary central nervous system lymphoma (PCNSL), the virus fosters immune evasion despite retained antigen presentation. The TME in EBV^+^ PCNSL is enriched in immunosuppressive macrophages and checkpoint molecules, setting it apart from EBV-negative cases, which exhibit more robust immune clearance [69]. Similarly, solid tumors, such as gastric carcinoma, exploit EBV-induced autocrine loops involving CSF1 and IL-10 to polarize CD163^+^ M2 macrophages. These M2 macrophages secrete matrix metalloproteinase 9 (MMP9), which not only exhausts T cells but also confers resistance to T cell receptor (TCR)-engineered T cell therapy. Interestingly, this resistance can be overcome by combining MMP9 inhibitors with adoptive T cell transfer, offering a promising strategy for therapeutic intervention [70]. In conclusion, EBV exploits macrophages as key mediators of immune evasion, either by skewing polarization toward protumorigenic M2 TAMs or by disrupting antitumor responses through M1 TAM dysregulation. Targeting the EBV–macrophage crosstalk represents a promising strategy for reversing TME immunosuppression, offering potential therapeutic avenues to reprogram the TME and enhance antitumor immunity (Fig. 4).

#### Other viruses

HPV infection is a major risk factor for HNSCC, with its carcinogenic effects largely mediated through the action of E6 and E7 oncoproteins, which degrade p53 and inactivate pRB, respectively. Beyond these canonical roles, HPV further subverts tumor immunity by orchestrating macrophage enrichment within the TME. Clinical evidence indicates that HPV^+^ tumors exhibit substantially denser intratumoral infiltration of CD68^+^ macrophages compared to HPV^−^ counterparts. This macrophage accumulation is correlated with advanced disease stage, increased recurrence risk, and reduced survival rates [71].

Mechanistically, HPV reprograms tumor cell metabolism through the action of E6/E7, inducing a Warburg-like metabolic shift that increases lactate production and stabilizes hypoxia-inducible factor-1α (HIF-1α). This metabolic reprogramming triggers up-regulation of macrophage MIF secretion from tumor cells, resulting in amplified accumulation of CD68^+^ macrophages [72]. These findings highlight the dual role of HPV in both promoting tumorigenesis and modulating the immune microenvironment by reshaping macrophage behavior.

Kaposi’s sarcoma-associated herpesvirus (KSHV) also exerts profound influence on macrophage function through both direct infection and co-infection paradigms, establishing immunosuppressive niches within the TME. Upon infecting macrophages, KSHV activates the IRE1α-XBP1 endoplasmic reticulum stress axis, which drives robust M2-like polarization. This is characterized by the up-regulation of CD163, activation of STAT3/STAT6 signaling, and the secretion of protumorigenic cytokines, including IL-10, vascular endothelial growth factor (VEGF), IL-6, and IL-8. Simultaneously, KSHV induces the expression of PD-L1, enforcing immune checkpoint inhibition and contributing to immune evasion [73]. In HPV-coinfected malignancies, such as cervical cancer, KSHV amplifies pathogenesis by up-regulating MIF and CXCR2 in tumor cells, creating feedforward loops that recruit and activate TAMs, despite down-regulation of HPV E6/E7 oncoproteins [74]. These mechanisms underscore the ability of KSHV to manipulate macrophage function and create a permissive TME for tumor progression.

In addition to these human pathogens, plant viruses also exploit macrophage reprogramming to overcome immune suppression in the TME. Tobacco mosaic virus (TMV), a plant-derived virus, directly binds to macrophage TLR4 receptors, activating MAPK/NF-κB signaling pathways that drive potent M1 polarization. This results in the production of proinflammatory cytokines and tumoricidal activity, which suppresses breast cancer metastasis in vivo [75]. On the other hand, Chikungunya virus (CHIKV), a human pathogen, targets immunosuppressive pathways in melanoma by blocking IL-10 receptor signaling, thus preventing M2 macrophage polarization. CHIKV also reverses T cell dysfunction by inhibiting STAT3, reestablishing antitumor immunity [76].

These complementary viral strategies, leveraging pathogen recognition receptors and cytokine network disruption, illustrate how viruses can counteract TAM immunosuppression. They highlight the potential for targeted reprogramming of TAM functionality as a strategy to overcome tumor immune evasion. This insight provides a foundation for developing novel viro-immunotherapeutic approaches that harness the ability of viruses to modulate macrophage polarization and enhance antitumor immunity.

### Other microbes and their effects on macrophages

Beyond bacteria and viruses, fungi and parasites substantially influence TAM behavior, with important implications for oncogenesis. The opportunistic fungal pathogen *Candida albicans* illustrates the context-dependent nature of microbial immunomodulation. Heat-killed *C. albicans* primes peritoneal macrophages in hepatoma models, enhancing endotoxin-sensitized tumoricidal activity [77]. In contrast, live *C. albicans* infection in oral cancer induces IL-17A/IL-17RA-driven M2-like TAM polarization, characterized by up-regulation of immune checkpoints such as PD-L1 and Galectin-9, ultimately accelerating tumor progression [78]. This dual role emphasizes how the pathogenic state of *C. albicans* can dictate macrophage functionality and its impact on tumor dynamics. Conversely, certain medicinal fungi exert potent immunomodulatory effects that favor antitumor immunity. *Hirsutella sinensis* fungus (*HSF*) has been shown to up-regulate the chemokine receptor CCRL2 in lung cancer, driving M1 macrophage polarization. This shift not only activates CD8^+^ T cell proliferation but also enhances their effector function, resulting in the suppression of tumor growth [79]. The ability of fungi like *HSF* to reprogram macrophage polarization toward an antitumor M1 phenotype underscores their therapeutic potential in cancer immunotherapy.

In addition to fungi, parasitic infections also modulate macrophage behavior with distinct outcomes in cancer. *Plasmodium* infection has been shown to inhibit hepatocellular carcinoma (HCC) progression by targeting TAMs. The parasite-derived metabolite hemozoin accumulates in TAMs, suppressing insulin-like growth factor 1 (IGF-1)-mediated phosphatidylinositol 3-kinase (PI3K)/MAPK signaling and down-regulating MMP9 expression. This disruption of angiogenesis limits tumor growth and metastasis [80]. These studies demonstrate how parasitic infections can manipulate macrophage functionality to exert antitumor effects, offering insights into novel therapeutic approaches involving host–pathogen interactions.

Beyond intact microorganisms, bioactive components derived from these microbes also serve as powerful immunomodulators that can reprogram TAMs. For example, the polysaccharide *Rhodzopus nigricans* (RPS-1) activates MAPK/NF-κB signaling in macrophages, leading to enhanced production of NO and TNF-α. This response suppresses colon tumor growth and synergizes with 5-fluorouracil (5-FU) chemotherapy [81]. Similarly, homogeneous *Polyporus* polysaccharide (HPP) repolarizes TAMs in bladder cancer via NF-κB/NLRP3 signaling, creating an anti-TME that indirectly induces autophagy in cancer cells by inhibiting the PI3K/Akt/mTOR pathway in macrophage–tumor cocultures [82,83]. Additionally, *Lachnum* polysaccharide (LEP) employs a dual mechanism: It directly triggers TLR4/NF-κB-mediated M1 polarization while indirectly promoting Th1-derived IFN-γ, which further reinforces macrophage reprogramming and reduces immunosuppressive cell infiltration [84]. These findings underscore the multifaceted ways in which fungal polysaccharides can influence macrophage polarization, offering a potential therapeutic strategy to counteract tumor-induced immune suppression.

In conclusion, across diverse microbial taxa—including bacteria, viruses, fungi, and parasites—a convergent signaling landscape governs macrophage reprogramming within the TME. Central to this convergence are innate immune sensors such as TLRs, which integrate pathogen-associated molecular patterns into downstream activation of NF-κB, MAPK, and STAT pathways. These cascades orchestrate transcriptional programs that determine macrophage fate, toggling between proinflammatory (M1 phenotype) and immunosuppressive (M2 phenotype) states. Microbial ligands such as LPS, capsular polysaccharides, or adhesins predominantly engage TLR2/TLR4 to drive NF-κB-dependent expression of IL-6, IL-12, and iNOS, enhancing antitumor immunity. Conversely, viral or dysbiotic microbial cues often exploit STAT3/STAT6 signaling to sustain IL-10 and TGF-β dominated environments that favor M2 polarization, angiogenesis, and immune evasion. In parallel, microbial metabolites—including short-chain fatty acids, TMAO, and fungal polysaccharides—reshape macrophage metabolism through PI3K–Akt–mTOR and HIF-1α-dependent axes, coupling metabolic adaptation with functional polarization. These intersecting pathways ultimately converge on macrophage plasticity, positioning TAMs as dynamic sensors that integrate microbial, metabolic, and host-derived cues to shape tumor progression. Recognizing these shared mechanistic nodes highlights therapeutic opportunities to selectively redirect macrophage programming—through modulation of TLR or STAT signaling—to restore antitumor immunity across microbiome-influenced cancers. Targeting these convergent signaling hubs not only offers a means to reawaken immunologically “cold” tumors but also provides a conceptual framework for integrating microbiome modulation with immunotherapy. By harnessing microbial cues or their synthetic mimetics to fine-tune macrophage polarization, it may become possible to reprogram the TME toward sustained immune activation and durable therapeutic response.

## Microbe-Based Strategies for Macrophage-Targeted Cancer Immunotherapy

### Engineered microbes for immune modulation

Engineered bacteria represent an emerging and promising therapeutic strategy for reprogramming the tumor immune microenvironment, offering novel possibilities for the treatment of cancer and related diseases. These bacteria, which have been specifically designed to modulate immune responses, enable the precise manipulation of TAMs and other immune cell subsets, driving immune activation and tumor eradication. One example of this innovative approach is complementary bacterial duo therapy, which combines the systemically administered short-lived *mp105*, a strain that combats tumors via direct oncolysis, TAM depletion, and CD4^+^ T cell immunity, with intratumorally injected glucose-sensing *m6001*. The latter selectively colonizes and lyses tumors through localized replication, amplifying therapeutic efficacy by combining tumor targeting with immune modulation [85].

Further advancing bacterial delivery systems, the “Trojan horse” platform encapsulates attenuated *Salmonella* in cryo-shocked macrophages treated with liquid nitrogen. This strategy enables the bacteria to retain bioactivity for delayed release inside the tumor while evading neutrophil-mediated clearance. Importantly, this method enhances tumor enrichment, converts M2 macrophages (TAMs) into M1 phenotype, and depletes Tregs, thus amplifying the antitumor immune response [86]. Another innovative approach involves IFN-γ-boosted *Salmonella* therapy, where cytokine-mediated blockade of neutrophils prolongs bacterial survival and simultaneously recruits M1 phenotype macrophages, along with CD4^+^ and CD8^+^ T cells, leading to enhanced tumor eradication [87].

The precision of engineered microbial therapies continues to evolve with the development of dual-engineered macrophage–microbe encapsulation systems. In this strategy, Arg-Gly-Asp-modified macrophages are outfitted with *Salmonella* secreting IFN-γ (*VNP-IFNγ*). These hybrid R-GEM/VNP-IFNγ cells leverage macrophage chemotaxis for tumor targeting, releasing bacteria that sustain antitumor effects while polarizing TAMs toward M1, maturing DCs, and expanding effector T cells across metastatic models [88]. This strategy exemplifies the potential of macrophage–microbe hybrid systems to enhance immune responses at the tumor site and resolve issues of spatial heterogeneity within the TME. In addition to these biohybrid systems, material-engineered bacteria such as the PA@LDH platform combine *Propionibacterium acnes* with cobalt–aluminum layered double hydroxides (LDHs) to enable pH-responsive tumor targeting. In this system, bacterial denitrification generates cytotoxic peroxynitrite (ONOO^−^), while cobalt ions inhibit superoxide dismutase, amplifying oxidative stress within the TME. At the same time, *P. acnes* drives the repolarization of M2 to M1 TAMs, sustaining a proinflammatory immune environment [89]. This combination of material engineering and microbial delivery substantially enhances immune modulation within the TME. Advancing the precision of tumor immunotherapy, Rhodobacter-based reporters exploit endogenous bacteriochlorophyll-a for optoacoustic imaging, enabling noninvasive mapping of macrophage activity-dependent spectral shifts. This technique provides a real-time understanding of TAM heterogeneity in 4T1/CT26 tumors and holds promise for integrating genetic engineering in theranostic applications [90]. At the forefront of engineered microbial systems, optogenetically controlled dual-strain platforms have been developed. In one such platform, a bacterium is engineered to produce GM-CSF in response to light, repolarizing M2 TAMs into M1 phenotypes, while another secretes outer membrane vesicle (OMV)-shielded small interfering RNA (siRNA) targeting SIRPα to block “don’t eat me” signals. This dual-engineered in situ bioproduction achieves a sustained inversion of the M1/M2 ratio (0.80 versus baseline), reducing SIRPα^+^ TAMs to 3.46%, outperforming conventional therapies [91]. In summary, these engineered microbial platforms highlight the tremendous potential of microbiome-based cancer interventions. They demonstrate how microbial therapies can overcome delivery barriers, resolve spatial heterogeneity in the TME, and durably reprogram TAMs to remodel the immunosuppressive microenvironment. By harnessing the precision of engineered microbes, these platforms offer novel and powerful strategies to enhance cancer immunotherapy and resolve current therapeutic limitations.

### Microbial nanomaterials and their role in immunotherapy

Microbial nanomaterials offer a powerful approach to precisely reprogram TAMs through engineered immunogenicity and stimuli-responsive delivery mechanisms. One notable class of nanomaterials is bacterial OMVs, which serve as versatile platforms for immunomodulation. For example, EGFR-targeted OMVs deliver TLR agonists to TAMs, promoting their polarization from an immunosuppressive M2 phenotype to a tumoricidal M1 phenotype in breast cancer, thereby enhancing antitumor immunity [92]. A more sophisticated application involves radiation-responsive PEG/Se@OMV-CD47nb, which sequentially unmasks CD47-blocking nanobodies and OMV immunogenicity. This system not only suppresses the “don’t eat me” signals mediated by CD47 but also drives phagocytic M1 polarization, thereby enhancing macrophage-mediated tumor cell clearance [93].

Complementary approaches leverage bacterial motility structures and charge-reversal polymers to prolong macrophage uptake and repolarize TAMs toward M1 phenotypes. Flagellar nanofiber vaccines, for example, exploit these properties to self-adjuvant M1 polarization, thereby suppressing melanoma metastasis [94]. Hybrid systems further enhance specificity and tumor targeting. For instance, liposome-conjugated *E. coli* engineered as hypoxia-triggered antibody factories are designed to locally block CD47 and activate TAM phagocytosis in hypoxic niches, directly reprogramming the TME to promote immune responses in the TME [95]. In another example, croconium-photosensitizer-conjugated *MG1655* strains target CDH17^+^ gastrointestinal tumors and release bacterial DNA upon phototherapy, activating the STING-dependent type-I interferon pathway in TAMs and facilitating synergistic antitumor immunity [96]. One of the most innovative approaches is the use of melittin-RADA32/metformin-coated *Clostridium novyi* spores, which reprogram glioblastoma microenvironments through HIF-1α suppression and sustained M1 polarization. This strategy not only reprograms TAMs to an antitumor phenotype but also enhances the therapeutic efficacy of cancer treatments by modulating the immune landscape in glioblastoma [97].

Beyond bacterial-derived nanomaterials, viral engineering platforms also harness innate immunogenicity and targeted delivery mechanisms to reprogram TAMs. For example, cowpea mosaic virus (CPMV) conjugated with CD206-targeting peptides (CPMV-CD206) selectively binds M2 TAMs, driving their conversion to tumoricidal M1 phenotypes and thereby suppressing melanoma progression. The efficacy of this approach, however, depends on factors beyond targeting specificity, such as the inflammatory microenvironment and the viral delivery efficiency [98]. Building upon this, CPMV-based in situ vaccination synergizes with CD47-blocking antibodies through dual-pathway activation: CPMV recruits and primes phagocytes, while CD47 blockade disables “don’t eat me” signals, enhancing macrophage-mediated tumor cell phagocytosis and bridging innate and adaptive immunity [99]. Most potently, oncolytic vaccine viruses (OVVs) engineered to secrete anti-CD47 nanobody–Fc fusions (*OVV-hCD47nb-G1*) achieve spatiotemporal precision in immune modulation. These viruses produce locally generated nanobodies that block CD47/SIRPα interactions on tumor cells while sparing red blood cells, simultaneously reprogramming TAMs to phagocytic states and enhancing immune cell infiltration. When combined with chimeric antigen receptor (CAR)-T cells, *OVV-hCD47nb-G1* overcomes immunosuppressive barriers by coordinating phagocytosis activation and T cell recruitment, ultimately inducing complete lymphoma regression [100].

### Application of OVs in tumor therapy

OVs are engineered or naturally occurring viruses that selectively infect and lyse cancer cells while sparing normal tissues. They exert their therapeutic effects through dual mechanisms: direct oncolysis of infected cancer cells and the stimulation of the host immune response to target both infected and neighboring uninfected tumor cells. Currently, OVs are being explored in combination with immune checkpoint inhibitors, chemotherapy, and radiation therapy to enhance their therapeutic efficacy. Oncolytic virotherapy gained considerable momentum with the 2015 U.S. Food and Drug Administration (FDA) approval of talimogene laherparepvec (T-VEC) for melanoma. T-VEC uses GM-CSF expression to stimulate antitumor immunity, marking a breakthrough in the clinical application of OVs [101]. Building on this foundation, engineered adenoviruses have been developed to precisely reprogram TAMs through innovative strategies. For example, *ADVNE/ADVPPE* adenoviruses arm pyroptosis-inducing transgenes, such as neutrophil elastase/porcine pancreatic elastase, to release HMGB1 from dying tumor cells. This alarmin binds TLR4 on TAMs, activating MyD88–NF-κB–NLRP3 signaling to drive M1 polarization, which enhances CD8^+^ T cell infiltration in CRC [102].

Another advanced strategy involves *SG635-SF*, an engineered adenovirus that incorporates a chimeric fiber for enhanced tropism and expresses SIRPα–Fc fusion proteins. This system blocks CD47 “don’t eat me” signals on ovarian cancer cells, unleashing TAM phagocytosis while increasing intratumoral macrophage density [103]. The most comprehensive approach thus far involves *AdV-SS* adenoviruses, which encode dual extracellular domains (SIRPα + Siglec-10) as secreted fusion proteins. These viruses simultaneously inhibit CD47 and CD24 immunosuppressive pathways, reprogramming TAMs toward antitumor phenotypes that up-regulate major histocompatibility complex (MHC)-I/II and activate CD8^+^ T cells across multiple carcinoma models, achieving localized checkpoint blockade without systemic toxicity [104].

Vaccinia-based vectors leverage their natural tropism for tumor hypoxia to deliver immunomodulatory payloads that remodel macrophage function. A replication-incompetent recombinant vaccinia virus expressing CD40 ligand (rVV40L) reprograms macrophages via dual mechanisms: inducing TNF-α-dependent tumoricidal activity in M1 phenotype macrophages against CD40^−^ tumors and “re-educating” M2-like macrophages to produce CXCL10 and IL-12, facilitating CD8^+^ T cell recruitment [105]. Complementarily, cytokine-armed variants have demonstrated enhanced efficacy. For example, a recombinant oncolytic vaccinia virus (VVL-GL7), coexpressing GM-CSF and IL-7, boosts macrophage infiltration and synergizes with PD-1 blockade in pancreatic cancer [106]. Furthermore, GM-CSF-armed vaccinia viruses eradicate tumors by promoting neutrophil and macrophage influx while establishing long-lasting antitumor memory [107]. Most innovatively, *AVL-lectin*-armed vaccinia triggers ROS-dependent IFN-γ release, systemically polarizing macrophages toward M1 phenotypes in ovarian cancer [108]. These strategies collectively reprogram TAMs, creating an effective bridge between innate and adaptive immunity to combat tumor progression.

Oncolytic herpes simplex viruses (oHSVs) also play a pivotal role in remodeling the TME by reprogramming TAM polarization toward antitumor phenotypes. Tumor-intrinsic herpes virus entry mediator (HVEM) suppresses metastasis in non-small cell lung cancer (NSCLC) by skewing TAMs toward M1 polarization via the GM-CSF/GM-CSFRα axis while inhibiting M2 differentiation [109]. In glioblastoma models, sustained intratumoral viral persistence, such as with the F-strain derivative rQNestin34.5v1 vector, correlates with prolonged macrophage and T cell infiltration, which is essential for effective antitumor immunity [110]. Furthermore, GM-CSF-armed oHSV2 (oHSV2-mGM) directly stimulates M1 polarization of macrophages in vitro and synergizes with αPD-1 therapy in vivo, achieving complete tumor remission by reversing post-ablation immunosuppression [111].

**Fig. 5.**
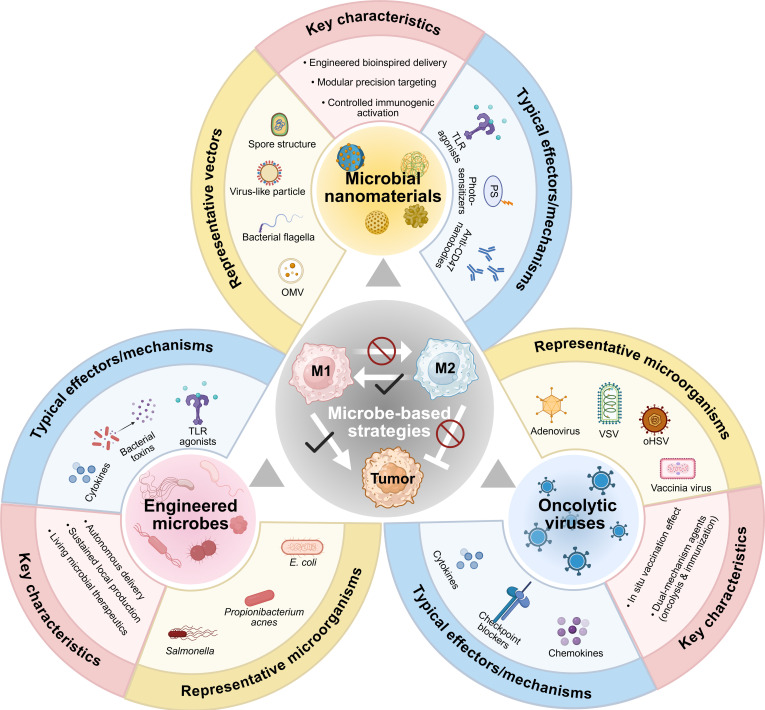
Engineered microbial systems for reprogramming TAMs in cancer immunotherapy. Synthetic biology and biomaterial-based approaches have enabled the development of advanced microbial systems capable of precisely targeting and reprogramming TAMs. Key strategies include engineered live bacteria such as *Salmonella* and *E. coli* that colonize tumors and secrete immunomodulatory agents to promote M1 polarization; microbial nanomaterials such as bacterial outer membrane vesicles and flagella-based structures that deliver TLR agonists or block don’t eat me signals like CD47; and OVs including adenovirus, vaccinia, and HSV armed with transgenes encoding cytokines, checkpoint inhibitors, or phagocytosis-promoting fusion proteins to stimulate antitumor immunity. These systems exhibit modular design features such as tumor-specific targeting, controlled immunogenic activation, and bioinspired delivery, enabling spatial and temporal control over TAM phenotype and function. Together, they represent a promising class of immunotherapies that can remodel the immunosuppressive TME.

Collectively, these findings underscore the capacity of OVs—ranging from adenoviruses and vaccinia viruses to herpes simplex vectors—to function as potent immunomodulatory tools that extend beyond direct oncolysis. By selectively reprogramming TAMs toward proinflammatory, antigen-presenting M1 phenotypes, these viral platforms effectively reshape the immunosuppressive TME, enhance cytotoxic T cell recruitment, and synergize with checkpoint blockade or other therapies. This dual capacity for tumor cell lysis and immune activation positions oncolytic virotherapy as a cornerstone strategy for macrophage-centered cancer immunotherapy (Fig. 5).

### Combination strategies with conventional cancer therapies

#### Combination with PTT/PDT

Photothermal therapy (PTT) and photodynamic therapy (PDT) are noninvasive strategies that induce ICD through localized heat or ROS production, thereby enhancing tumor immunogenicity. However, limited tumor penetration and insufficient immune activation often compromise therapeutic outcomes. Combining these modalities with TAM reprogramming strategies markedly enhances ICD and reverses immunosuppression. For instance, a tumor-targeted bacterial biohybrid (named eVNP@AuNFs) down-regulates CD47 and heat shock protein 90 (HSP90) via short hairpin RNA (shRNA) plasmids to block “don’t eat me” signals, while its gold nanoparticle component mediates second near-infrared (NIR-II)-triggered PTT to induce ICD markers like calreticulin, thereby synergistically boosting macrophage phagocytosis and systemic immunity in 4T1 tumors [112]. Similarly, the B.b@QDs system exploits *Bifidobacterium* bifidum’s hypoxia tropism to penetrate tumors and directly polarize TAMs toward M1 phenotypes; concurrently, Ag₂S quantum dots enable PTT-induced ICD, overcoming tumor heterogeneity in breast cancer models [113]. Expanding this approach, CPMV/dendron hybrids deliver photosensitizers specifically to immunosuppressive TAMs and melanoma cells, depleting protumoral macrophages while inducing tumor-selective PDT [114]. Furthermore, a bioinspired coronavirus-like nanocarrier (named SDN) achieves mitochondria-specific PDT through cationic spike-mediated targeting while generating oxygen via MnO₂ decomposition to alleviate hypoxia and amplify macrophage-associated immune responses [115]. Notably, self-illuminating systems such as OVV-Luc@Ce6 (OV@C) leverage OV-induced luminescence to activate Ce6-PDT without external light, synergizing with viral immunotherapy to polarize TAMs and convert immunologically “cold” colorectal tumors [116]. Lastly, probiotic-derived EcNZ/F@Au utilizes NIR-triggered transformation into bacterial ghosts for spatiotemporal release of 5-FU and zoledronic acid (ZOL), where ZOL reprograms TAMs to M1 to synergize with photothermal chemotherapy [117].

#### Combination with chemotherapy

Chemotherapy remains a cornerstone of cancer treatment, but its efficacy is often undermined by poor tumor specificity and the immunosuppressive TME dominated by M2-like TAMs. To address these challenges, bacterial platforms have been integrated with chemotherapeutics to achieve targeted delivery and immune reprogramming. For example, DOX@Bio-Bac incorporates *Staphylococcus aureus* walls into liposomes to mimic bacterial immunogenicity, effectively re-educating TAMs toward M1 phenotypes while delivering doxorubicin to inhibit HCC growth and metastasis. In glioblastoma, the MEK inhibitor trametinib blocks TNF-α secretion from microglia and TAMs, which otherwise restricts oncolytic HSV-1 replication; this combination rescues MAPK pathway reactivation and enhances CD8^+^ T cell immunity [118]. Clinically, the oncolytic Newcastle disease virus MEDI5395 (expressing GM-CSF) combined with anti-PD-L1 (durvalumab) reprograms TAMs and promotes T cell infiltration, yielding partial responses in patients with high baseline PD-L1/CD8^+^ levels despite neutralizing antibody development [119].

#### Combination with radiation/ultrasound therapy

Radiotherapy and focused ultrasound induce immunogenic tumor cell death through DNA damage or mechanical/thermal stress, effects amplified by TAM modulation. Specifically, Ec@DIG-GVs express ultrasound-visible gas vesicles to guide hyperthermic HIFU, triggering IFN-γ production that repolarizes TAMs to M1, matures DCs, and synergizes with acid-released doxorubicin for chemo-immunotherapy [120]. Similarly, radioiodine-labeled inactivated bacteria (^131^I-VNP) achieve sustained internal radiotherapy while activating the cGAS-STING pathway to repolarize TAMs and promote DC maturation; this elicits systemic immunity that, when combined with systemic checkpoint blockade therapy (αPD-L1), controls distant tumors [121]. Complementarily, high-dose hypofractionated stereotactic body radiotherapy (SBRT) with oncolytic vaccinia virus triggers tumor necroptosis and damage-associated molecular pattern (DAMP) release, shifting TAMs toward M1 phenotypes, depleting regulatory T cells, and expanding tumor-infiltrating lymphocytes for abscopal effects [122].

#### Combination with immunotherapy

Immunotherapy aims to activate the host immune system against tumors, but its efficacy is often limited by immunosuppressive TME, particularly TAMs. To address this, engineered *Salmonella* typhimurium strains (SAMphIF/SAMpmIF) secrete bioactive IL-15/FlaB fusion proteins, which repolarize TAMs from M2 to M1 phenotypes while activating CD4^+^/CD8^+^ T, NK, and NKT cells in MC38 and CT26 tumors; furthermore, combining these bacteria with anti-PD-L1 robustly suppresses metastasis and induces long-term immune memory [123]. Similarly, macrophage-mediated delivery of *Salmonella* (VNP-PD1nb) reduces systemic toxicity while enabling tumor-specific release of anti-PD-1 nanobodies, reactivating the TME by increasing immune infiltration and M1 phenotype TAM polarization [124]. Expanding on bacterial precision, PD-1@EcM employs nonpathogenic *E. coli* with acid-responsive gene circuits to produce anti-PD-1 scFv in residual tumors post-thermal ablation, repolarizing TAMs and priming CD8^+^ T cells in HCC models [125]. Beyond live bacteria, BDVs-Neo vaccines—comprising bacterial-derived vesicles presenting neoantigens—recruit DCs via sustained GM-CSF/LPS release; when combined with anti-PD-1, they enhance tumor-infiltrating T cell activation and prevent relapse [126]. Critically, attenuated *Brucella* (Bm∆vjbR) remodels the TME by polarizing TAMs to M1 and boosting CD8^+^ T cell activity, thereby overcoming resistance to CAR-T therapy in colon adenocarcinoma and achieving 100% survival [127]. For adoptive cell therapy, Mø@bac attaches bacteria to infused macrophages via adhesive nanocoatings, providing durable stimulation to maintain antitumor phenotypes in vivo while repolarizing endogenous TAMs in 4T1 tumors [128]. Finally, oncolytic vaccinia virus VVL-GL21—armed with GM-CSF/IL-21—shifts TAMs from M2 to M1, matures DCs, and synergizes with anti-PD-1 to regress pancreatic tumors systemically, demonstrating abscopal effects against lung/colon cancers [129].

These multifaceted approaches collectively enhance ICD while subverting TAM-mediated immunosuppression through distinct yet complementary mechanisms: PDT/PTT combinations amplify “eat me” signals and oxygen-dependent immunity; chemotherapy integrations overcome macrophage-driven drug resistance; and radiation/ultrasound therapies leverage DAMPs and STING pathway activation to generate systemic responses. Critically, the strategic integration of TAM-focused interventions with immunotherapy leverages engineered biological vectors (bacteria, viruses) and nanomaterials—capitalizing on bacterial tumor tropism, viral oncolytic activity, and biomaterial precision—to reverse immunosuppressive barriers. This dual targeting of innate (TAM reprogramming) and adaptive immunity not only provides spatiotemporal control and microenvironmental responsiveness but also unlocks potent systemic antitumor immunity with durable memory, unequivocally positioning TAM modulation as the cornerstone of next-generation combination therapies (Table 1).

**Table 1.** Synergistic strategies combining microbe–TAM targeting with conventional cancer therapies

Combined therapy	Microbial vector/platform	Key mechanism of action	Outcome	Ref.
PTT	eVNP@AuNFs	NIR-II-triggered PTT; down-regulation of CD47/HSP90 via shRNA plasmids	Boosting of macrophage phagocytosis and systemic immunity in 4T1 tumors	[112]
PTT	B.b@QDs	Tumor penetration and M1 TAM polarization; PTT-induced ICD	Promoting M1 TAM polarization and antitumor immunity in breast cancer models	[113]
PDT	CPMV/dendron hybrids	Targeted photosensitizer delivery to TAMs and tumor cells	Depletion of protumoral macrophages and tumor cell death	[114]
PDT	SDN nanocomplexes	Mitochondria-specific PDT; oxygen generation via MnO_2_ decomposition	Amplification of macrophage-associated immune responses	[115]
PDT	OVV-Luc@Ce6	Virus-induced luminescence for external light-independent PDT activation	Enhancing DC maturation, macrophage polarization, and conversion of immunologically cold tumors	[116]
Photothermal chemotherapy	EcNZ/F@Au	NIR-triggered transformation into bacterial ghosts for spatiotemporal release of 5-FU and ZOL	Enhancing polarization of TAMs toward M1 and production of proinflammatory cytokines	[117]
Chemotherapy	DOX@Bio-Bac	*S. aureus* wall-coated immunogenicity mimicry; TAM re-education to M1; doxorubicin delivery	Inhibiting HCC growth and metastasis	[118]
Chemotherapy	Trametinib + oHSV	Expression of GM-CSF; TAM reprogramming; T cell infiltration promotion	Achievement of partial responses in high PD-L1/CD8^+^patients	[119]
Ultrasound therapy	Ec@DIG-GVs	Ultrasound-guided HIFU hyperthermia; IFN-γ production induction	Repolarizing TAMs to M1, enhancing DC maturation	[120]
Radiotherapy	¹³¹I-VNP	Sustained internal radiotherapy; cGAS-STING pathway activation	Repolarizing TAMs, promoting DC maturation, and eliciting systemic immunity to control distant tumors	[121]
Radiotherapy	SBRT + oncolytic vaccinia	Radiation-induced tumor necroptosis; DAMPs release	Shifting TAMs to M1, depleting Tregs, and expanding tumor-infiltrating lymphocytes for abscopal effects	[122]
Immunotherapy	SAMphIF/SAMpmIF + anti-PD-L1	Secretion of IL-15/FlaB fusion proteins; TAM repolarization; NK/NKT cell activation	Suppressing metastasis; inducing long-term immune memory in MC38/CT26 tumors	[123]
Immunotherapy	VNP-PD1nb	Macrophage-mediated delivery of *Salmonella;* tumor-specific nanobody release	Increasing immune infiltration and M1 phenotype TAM polarization, reducing systemic toxicity	[124]
Immunotherapy	PD-1@EcM	Acid-responsive production of anti-PD-1 scFv	Repolarizing TAMs and priming CD8^+^ T cells in HCC	[125]
Immunotherapy	BDVs-Neo + anti-PD-1	Neoantigen presentation; DC recruitment via GM-CSF/LPS release	Enhancing T cell activation and preventing relapse	[126]
Immunotherapy	Bm∆vjbR + CAR-T	TME remodeling through TAM polarization; CD8^+^ T cell activity boost	Overcoming resistance to CAR-T therapy in colon adenocarcinoma	[127]
Immunotherapy	Mø@bac	Bacterial attachment via nanocoatings for sustained stimulation	Maintaining antitumor phenotypes and repolarizing endogenous TAMs in 4T1 tumors	[128]
Immunotherapy	VVL-GL21 + anti-PD-1	Armed with GM-CSF/IL-21 shifting TAMs from M2 to M1 and maturing DCs	Regressing pancreatic tumors systemically and demonstrating abscopal effects	[129]

## Challenges and Future Directions

Despite the growing promise of targeting the microbe–TAM axis for cancer immunotherapy, several formidable challenges remain that hinder both mechanistic understanding and clinical translation. A primary obstacle lies in the profound spatial and temporal heterogeneity of the intratumoral microbiota. Distinct microbial niches have been observed within tumor tissues, with specific microbes such as *F. nucleatum* preferentially enriched in hypoxic zones [42]. Additionally, microbial profiles exhibit marked differences between primary and metastatic lesions and vary across tumor molecular subtypes [47]. These variations are further influenced by host factors such as diet, antibiotic exposure, and circadian rhythms, which complicate the identification of causal microbe–TAM interactions and hinder the development of universal biomarkers for patient stratification [24]. To address this challenge, future studies should prioritize the spatiotemporal mapping of microbe–TAM interactions using advanced tools such as spatial multi-omics, high-resolution intravital imaging, and optoacoustic reporters. These methodologies will help decipher the dynamic and context-dependent relationships within specific tumor niches, facilitating a more precise understanding of microbial influence on TAM function.

Secondly, the mechanistic complexity of microbe-mediated TAM modulation remains a formidable hurdle. Microbes employ diverse and often redundant strategies to reprogram macrophage behavior, including microbial metabolites, pathogen-associated molecular patterns, and epigenetic modifiers [61,92,130]. A key future direction involves dissecting these conserved pathways to identify core effector molecules and their receptors on TAMs. Understanding how these signals integrate with TME factors, such as hypoxia and cytokines, will be critical for designing targeted interventions that selectively modulate macrophage function and improve therapeutic efficacy.

Thirdly, safety and specificity concerns persist with engineered microbial therapeutics. While attenuated live bacteria and OVs exhibit potent immunomodulatory properties, their clinical use is constrained by the risks of uncontrolled replication, systemic toxicity, or rapid clearance by the host immune system [131]. Microbial nanomaterials offer improved safety profiles, yet achieving tumor-selective delivery while avoiding off-target immune activation remains a critical challenge [132]. The future of engineered microbial therapies lies in precision microbial engineering, including the development of “smart” microbes with inducible gene circuits (e.g., light-, pH-, or hypoxia-sensitive) integrated with biomaterials for controlled, localized immunomodulation. Such systems would minimize systemic exposure while enhancing therapeutic efficacy at the tumor site.

Fourthly, there exists a notable translational gap between preclinical models and human cancer. Most studies rely on murine models or tumor-derived cell lines, which poorly recapitulate the diversity of the human microbiome, immune landscape, and TME. Technical limitations also hinder the detection and functional validation of low-biomass intratumoral microbes in human specimens. Bridging this gap will require the establishment of more human-relevant models, such as humanized mice or 3-dimensional (3D) organoid–immune–microbe cocultures, and the application of highly sensitive, contamination-resistant sequencing technologies to validate findings in human cohorts [133,134].

Finally, the integration of microbe–TAM-targeted strategies into existing therapeutic regimens introduces further complexity. For instance, antibiotic use may antagonize bacteria-based therapies, while the optimal timing, dosing, and combination with immune checkpoint blockade, chemotherapy, or radiotherapy remain poorly defined. Predictive biomarkers to guide these combinations are also lacking. Future clinical translation will depend on rationally designed combination trials and the development of biomarker-driven strategies, based on microbial signatures or TAM phenotypes, for patient stratification [135,136]. Furthermore, exploring microbiome-personalized immunotherapy—such as using defined probiotic consortia or fecal microbiota transplantation (FMT) to modulate the TME—represents a promising frontier to overcome therapy resistance and improve clinical outcomes [137–139].

Addressing these challenges will require interdisciplinary collaboration across microbiology, immunology, oncology, and systems biology. The path forward involves a concerted effort to map, understand, engineer, and clinically validate these complex interactions to realize the full potential of microbe–TAM-targeted therapies. This holistic approach will help overcome current limitations and pave the way for the next generation of precision immunotherapies (Table S1).

## Conclusion

The intricate interplay between tumor-resident microbes and TAMs represents a critical regulatory axis within the TME, exerting profound effects on immune responses, tumor progression, and therapeutic outcomes. As explored in this review, microbes—including bacteria, viruses, fungi, and their molecular derivatives—modulate multiple facets of TAM biology, ranging from polarization and metabolism to secretory activity and phagocytic function. These interactions dictate whether the immune microenvironment promotes or suppresses tumor growth, making the microbe–TAM axis a compelling target for therapeutic intervention.

Recent strategies designed to harness this axis—through engineered microbes, microbial nanomaterials, OVs, and metabolite-based interventions—have shown impressive preclinical potential to reprogram TAMs and reverse immunosuppression. By leveraging the inherent biology of the TME, these approaches stimulate innate immunity while bridging adaptive immune responses, often resulting in systemic and durable antitumor effects. Notably, these interventions can selectively modulate macrophage function in ways that traditional therapies may not, offering the unique advantage of reprogramming the TME’s immune landscape to favor tumor suppression.

Despite the notable progress made, challenges remain in fully understanding and exploiting the microbe–TAM axis. Issues of spatial and temporal heterogeneity, mechanistic complexity, safety concerns, and the translational gap between preclinical models and human cancer must still be addressed. However, the convergence of advanced technologies in spatial biology, synthetic biology, and nanomedicine is equipping researchers with the tools needed to overcome these hurdles. High-resolution imaging, precise gene editing, and engineered microbial systems hold promise in refining our understanding of this dynamic interaction.

Targeting the microbe–TAM dialogue goes beyond conventional immunotherapy paradigms, offering a novel and powerful approach to reprogramming the TME. As research in this field continues to mature, it holds the potential to revolutionize cancer immunotherapy, providing more personalized and effective treatments. This evolving approach could usher in a new era of targeted therapies that not only improve clinical outcomes but also redefine how we approach cancer treatment, making it more adaptable and responsive to individual patient profiles.

## Data Availability

No data were used for the research described in the article.

## References

[1] Xun Z, Ding X, Zhang Y, Zhang B, Lai S, Zou D, Zheng J, Chen G, Su B, Han L, et al. Reconstruction of the tumor spatial microenvironment along the malignant-boundary-nonmalignant axis. Nat Commun. 2023;14(1):933. 36806082 10.1038/s41467-023-36560-7 PMC9941488

[2] Arner EN, Rathmell JC. Metabolic programming and immune suppression in the tumor microenvironment. Cancer Cell. 2023;41(3):421–433. 36801000 10.1016/j.ccell.2023.01.009 PMC10023409

[3] Missiaen R, Lesner NP, Simon MC. HIF: A master regulator of nutrient availability and metabolic cross-talk in the tumor microenvironment. EMBO J. 2023;42(6): Article e112067. 36808622 10.15252/embj.2022112067 PMC10015374

[4] Sibai M, Cervilla S, Grases D, Musulen E, Lazcano R, Mo CK, Davalos V, Fortian A, Bernat A, Romeo M, et al. The spatial landscape of cancer hallmarks reveals patterns of tumor ecological dynamics and drug sensitivity. Cell Rep. 2025;44: Article 115229. 39864059 10.1016/j.celrep.2024.115229

[5] Zhang L, Xu J, Zhou S, Yao F, Zhang R, You W, Dai J, Yu K, Zhang Y, Baheti T, et al. Endothelial DGKG promotes tumor angiogenesis and immune evasion in hepatocellular carcinoma. J Hepatol. 2024;80:82–98. 37838036 10.1016/j.jhep.2023.10.006

[6] Xu J, Ding L, Mei J, Hu Y, Kong X, Dai S, Bu T, Xiao Q, Ding K. Dual roles and therapeutic targeting of tumor-associated macrophages in tumor microenvironments. Signal Transduct Target Ther. 2025;10:268. 40850976 10.1038/s41392-025-02325-5 PMC12375796

[7] Pyonteck SM, Akkari L, Schuhmacher AJ, Bowman RL, Sevenich L, Quail DF, Olson OC, Quick ML, Huse JT, Teijeiro V, et al. CSF-1R inhibition alters macrophage polarization and blocks glioma progression. Nat Med. 2013;19(10):1264–1272. 24056773 10.1038/nm.3337 PMC3840724

[8] Zhang X, Yue L, Cao L, Liu K, Yang S, Liang S, Liu L, Zhao C, Wu D, Wang Z, et al. Tumor microenvironment-responsive macrophage-mediated immunotherapeutic drug delivery. Acta Biomater. 2024;186:369–382. 39097127 10.1016/j.actbio.2024.07.042

[9] Martinenaite E, Lecoq I, Aaboe-Jorgensen M, Ahmad SM, Perez-Penco M, Glockner HJ, Chapellier M, Lara de la Torre L, Olsen LR, Romer AMA, et al. Arginase-1-specific T cells target and modulate tumor-associated macrophages. J Immunother Cancer. 2025;13(1): Article e009930. 39880485 10.1136/jitc-2024-009930 PMC11781113

[10] Sun L, Kees T, Almeida AS, Liu B, He XY, Ng D, Han X, Spector DL, McNeish IA, Gimotty P, et al. Activating a collaborative innate-adaptive immune response to control metastasis. Cancer Cell. 2021;39(10):1361–1374.e9. 34478639 10.1016/j.ccell.2021.08.005 PMC8981964

[11] Jiang SS, Xie YL, Xiao XY, Kang ZR, Lin XL, Zhang L, Li CS, Qian Y, Xu PP, Leng XX, et al. Fusobacterium nucleatum-derived succinic acid induces tumor resistance to immunotherapy in colorectal cancer. Cell Host Microbe. 2023;31:781–797.e789. 37130518 10.1016/j.chom.2023.04.010

[12] Li N, Zhou H, Holden VK, Deepak J, Dhilipkannah P, Todd NW, Stass SA, Jiang F. Streptococcus pneumoniae promotes lung cancer development and progression. iScience. 2023;26: Article 105923. 36685035 10.1016/j.isci.2022.105923 PMC9852931

[13] Yugawa T, Kiyono T. Molecular mechanisms of cervical carcinogenesis by high-risk human papillomaviruses: Novel functions of E6 and E7 oncoproteins. Rev Med Virol. 2009;19(2):97–113. 19156753 10.1002/rmv.605

[14] Sivan A, Corrales L, Hubert N, Williams JB, Aquino-Michaels K, Earley ZM, Benyamin FW, Lei YM, Jabri B, Alegre ML, et al. Commensal Bifidobacterium promotes antitumor immunity and facilitates anti-PD-L1 efficacy. Science. 2015;350(6264):1084–1089. 26541606 10.1126/science.aac4255 PMC4873287

[15] Nie F, Zhang J, Tian H, Zhao J, Gong P, Wang H, Wang S, Yang P, Yang C. The role of CXCL2-mediated crosstalk between tumor cells and macrophages in Fusobacterium nucleatum-promoted oral squamous cell carcinoma progression. Cell Death Dis. 2024;15(4):277. 38637499 10.1038/s41419-024-06640-7 PMC11026399

[16] Ginhoux F, Jung S. Monocytes and macrophages: Developmental pathways and tissue homeostasis. Nat Rev Immunol. 2014;14:392–404. 24854589 10.1038/nri3671

[17] Zhang H, Yu Y, Zhou L, Ma J, Tang K, Xu P, Ji T, Liang X, Lv J, Dong W, et al. Circulating tumor microparticles promote lung metastasis by reprogramming inflammatory and mechanical niches via a macrophage-dependent pathway. Cancer Immunol Res. 2018;6(9):1046–1056. 30002156 10.1158/2326-6066.CIR-17-0574

[18] Li X, Yao W, Yuan Y, Chen P, Li B, Li J, Chu R, Song H, Xie D, Jiang X, et al. Targeting of tumour-infiltrating macrophages via CCL2/CCR2 signalling as a therapeutic strategy against hepatocellular carcinoma. Gut. 2017;66(1):157–167. 26452628 10.1136/gutjnl-2015-310514

[19] Wenes M, Shang M, Di Matteo M, Goveia J, Martin-Perez R, Serneels J, Prenen H, Ghesquiere B, Carmeliet P, Mazzone M. Macrophage metabolism controls tumor blood vessel morphogenesis and metastasis. Cell Metab. 2016;24(5):701–715. 27773694 10.1016/j.cmet.2016.09.008

[20] Cheng M, Chen S, Li K, Wang G, Xiong G, Ling R, Zhang C, Zhang Z, Han H, Chen Z, et al. CD276-dependent efferocytosis by tumor-associated macrophages promotes immune evasion in bladder cancer. Nat Commun. 2024;15:2818. 38561369 10.1038/s41467-024-46735-5 PMC10985117

[21] Trotta R, Rivis S, Zhao S, Orban MP, Trusso Cafarello S, Charatsidou I, Pozniak J, Dehairs J, Vanheer L, Pulido Vicuna CA, et al. Activated t cells break tumor immunosuppression by macrophage reeducation. Cancer Discov. 2025;15(7):1410–1436. 40094380 10.1158/2159-8290.CD-24-0415 PMC12223510

[22] Kallis MP, Maloney C, Blank B, Soffer SZ, Symons M, Steinberg BM. Pharmacological prevention of surgery-accelerated metastasis in an animal model of osteosarcoma. J Transl Med. 2020;18(1):183. 32354335 10.1186/s12967-020-02348-2 PMC7193344

[23] Matusiak M, Hickey JW, van IJzendoorn DGP, Lu G, Kidziński L, Zhu S, Colburg DRC, Luca B, Phillips DJ, Brubaker SW. Spatially segregated macrophage populations predict distinct outcomes in colon cancer. *Cancer Discov*. 2024;14:1418–1439. 10.1158/2159-8290.CD-23-1300 PMC11294822 38552005

[24] Knudsen-Clark AM, Mwangi D, Cazarin J, Morris K, Baker C, Hablitz LM, McCall MN, Kim M, Altman BJ. Circadian rhythms of macrophages are altered by the acidic tumor microenvironment. EMBO Rep. 2024;25(11):5080–5112. 39415049 10.1038/s44319-024-00288-2 PMC11549407

[25] Locati M, Curtale G, Mantovani A. Diversity, mechanisms, and significance of macrophage plasticity. Annu Rev Pathol. 2020;15:123–147. 31530089 10.1146/annurev-pathmechdis-012418-012718 PMC7176483

[26] Chen R, Wang J, Dai X, Wu S, Huang Q, Jiang L, Kong X. Augmented PFKFB3-mediated glycolysis by interferon-gamma promotes inflammatory M1 polarization through the JAK2/STAT1 pathway in local vascular inflammation in Takayasu arteritis. Arthritis Res Ther. 2022;24(1):266. 36510278 10.1186/s13075-022-02960-1 PMC9743547

[27] Shapouri-Moghaddam A, Mohammadian S, Vazini H, Taghadosi M, Esmaeili SA, Mardani F, Seifi B, Mohammadi A, Afshari JT, Sahebkar A. Macrophage plasticity, polarization, and function in health and disease. J Cell Physiol. 2018;233(9):6425–6440. 10.1002/jcp.26429 PMC13482560 29319160

[28] Deng L, Ouyang B, Tang W, Wang N, Yang F, Shi H, Zhang Z, Yu H, Chen M, Wei Y, et al. Icariside II modulates pulmonary fibrosis via PI3K/Akt/beta-catenin pathway inhibition of M2 macrophage program. Phytomedicine. 2024;130: Article 155687. 38759312 10.1016/j.phymed.2024.155687

[29] Woo SJ, Kim Y, Kang HJ, Jung H, Youn DH, Hong Y, Lee JJ, Hong JY. Tuberculous pleural effusion-induced Arg-1^+^ macrophage polarization contributes to lung cancer progression via autophagy signaling. Respir Res. 2024;25(1):198. 38720340 10.1186/s12931-024-02829-8 PMC11077851

[30] Zhang Q, Sioud M. Tumor-associated macrophage subsets: Shaping polarization and targeting. Int J Mol Sci. 2023;24(8):7493. 37108657 10.3390/ijms24087493 PMC10138703

[31] Chen W, Chen M, Hong L, Xiahenazi A, Huang M, Tang N, Yang X, She F, Chen Y. M2-like tumor-associated macrophage-secreted CCL2 facilitates gallbladder cancer stemness and metastasis. Exp Hematol Oncol. 2024;13(1):83. 39138521 10.1186/s40164-024-00550-2 PMC11320879

[32] Zeng XY, Xie H, Yuan J, Jiang XY, Yong JH, Zeng D, Dou YY, Xiao SS. M2-like tumor-associated macrophages-secreted EGF promotes epithelial ovarian cancer metastasis via activating EGFR-ERK signaling and suppressing lncRNA LIMT expression. Cancer Biol Ther. 2019;20(7):956–966. 31062668 10.1080/15384047.2018.1564567 PMC6606001

[33] Roy S, Bag AK, Dutta S, Polavaram NS, Islam R, Schellenburg S, Banwait J, Guda C, Ran S, Hollingsworth MA, et al. Macrophage-derived neuropilin-2 exhibits novel tumor-promoting functions. Cancer Res. 2018;78(19):5600–5617. 30111533 10.1158/0008-5472.CAN-18-0562 PMC6168405

[34] Butkute A, Baltramonaitis M, Malmige S, Darinskas A, Pasukoniene V, Mlynska A. Targeting stemness pathways modulates macrophage polarization and reprograms the tumor microenvironment. Front Immunol. 2025;16:1513404. 40160820 10.3389/fimmu.2025.1513404 PMC11950675

[35] Li J, Ma A, Zhang R, Chen Y, Bolyard C, Zhao B, Wang C, Pich T, Li W, Sun N, et al. Targeting metabolic sensing switch GPR84 on macrophages for cancer immunotherapy. Cancer Immunol Immunother. 2024;73(3):52. 38349405 10.1007/s00262-023-03603-3 PMC10864225

[36] Nejman D, Livyatan I, Fuks G, Gavert N, Zwang Y, Geller LT, Rotter-Maskowitz A, Weiser R, Mallel G, Gigi E, et al. The human tumor microbiome is composed of tumor type-specific intracellular bacteria. Science. 2020;368(6494):973–980. 32467386 10.1126/science.aay9189 PMC7757858

[37] Smith A, Pierre JF, Makowski L, Tolley E, Lyn-Cook B, Lu L, Vidal G, Starlard-Davenport A. Distinct microbial communities that differ by race, stage, or breast-tumor subtype in breast tissues of non-Hispanic Black and non-Hispanic White women. Sci Rep. 2019;9(1):11940. 31420578 10.1038/s41598-019-48348-1 PMC6697683

[38] Gao Z, Guo B, Gao R, Zhu Q, Qin H. Microbiota disbiosis is associated with colorectal cancer. Front Microbiol. 2015;6:20. 25699023 10.3389/fmicb.2015.00020 PMC4313696

[39] Flemer B, Lynch DB, Brown JM, Jeffery IB, Ryan FJ, Claesson MJ, O’Riordain M, Shanahan F, O’Toole PW. Tumour-associated and non-tumour-associated microbiota in colorectal cancer. Gut. 2017;66(4):633–643. 26992426 10.1136/gutjnl-2015-309595 PMC5529966

[40] Yu G, Gail MH, Consonni D, Carugno M, Humphrys M, Pesatori AC, Caporaso NE, Goedert JJ, Ravel J, Landi MT. Characterizing human lung tissue microbiota and its relationship to epidemiological and clinical features. Genome Biol. 2016;17(1):163. 27468850 10.1186/s13059-016-1021-1 PMC4964003

[41] Wang X, Xiao T, Lu M, Wu Z, Chen L, Zhang Z, Lu W. Lower respiratory tract microbiome and lung cancer risk prediction in patients with diffuse lung parenchymal lesions. Front Cell Infect Microbiol. 2024;14:1410681. 39185086 10.3389/fcimb.2024.1410681 PMC11341542

[42] Battaglia TW, Mimpen IL, Traets JJH, van Hoeck A, Zeverijn LJ, Geurts BS, de Wit GF, Noe M, Hofland I, Vos JL, et al. A pan-cancer analysis of the microbiome in metastatic cancer. Cell. 2024;187(9):2324–2335.e19. 38599211 10.1016/j.cell.2024.03.021

[43] Scholze J. Angiotensin II receptor antagonists clinical relevance. Internist. 1996;37:636–642. 8767999

[44] Nakkarach A, Foo HL, Song AA, Mutalib NEA, Nitisinprasert S, Withayagiat U. Anti-cancer and anti-inflammatory effects elicited by short chain fatty acids produced by Escherichia coli isolated from healthy human gut microbiota. Microb Cell Factories. 2021;20(1):36. 10.1186/s12934-020-01477-z PMC7863513 33546705

[45] Wang H, Rong X, Zhao G, Zhou Y, Xiao Y, Ma D, Jin X, Wu Y, Yan Y, Yang H, et al. The microbial metabolite trimethylamine N-oxide promotes antitumor immunity in triple-negative breast cancer. Cell Metab. 2022;34(4):581–594.e8. 35278352 10.1016/j.cmet.2022.02.010

[46] Fu A, Yao B, Dong T, Chen Y, Yao J, Liu Y, Li H, Bai H, Liu X, Zhang Y, et al. Tumor-resident intracellular microbiota promotes metastatic colonization in breast cancer. Cell. 2022;185(8):1356–1372.e26. 35395179 10.1016/j.cell.2022.02.027

[47] Zhao F, An R, Ma Y, Yu S, Gao Y, Wang Y, Yu H, Xie X, Zhang J. Integrated spatial multi-omics profiling of Fusobacterium nucleatum in breast cancer unveils its role in tumour microenvironment modulation and cancer progression. Clin Transl Med. 2025;15: Article e70273. 40070022 10.1002/ctm2.70273 PMC11897063

[48] Ma J, Huang L, Hu D, Zeng S, Han Y, Shen H. The role of the tumor microbe microenvironment in the tumor immune microenvironment: Bystander, activator, or inhibitor? J Exp Clin Cancer Res. 2021;40:327. 34656142 10.1186/s13046-021-02128-w PMC8520212

[49] Zegarra Ruiz DF, Kim DV, Norwood K, Saldana-Morales FB, Kim M, Ng C, Callaghan R, Uddin M, Chang LC, Longman RS, et al. Microbiota manipulation to increase macrophage IL-10 improves colitis and limits colitis-associated colorectal cancer. Gut Microbes. 2022;14:2119054. 36062329 10.1080/19490976.2022.2119054 PMC9450902

[50] Yang Y, Li L, Xu C, Wang Y, Wang Z, Chen M, Jiang Z, Pan J, Yang C, Li X, et al. Cross-talk between the gut microbiota and monocyte-like macrophages mediates an inflammatory response to promote colitis-associated tumourigenesis. Gut. 2020;70(8):1495–1506. 33122176 10.1136/gutjnl-2020-320777 PMC8292576

[51] Utispan K, Pugdee K, Koontongkaew S. Porphyromonas gingivalis lipopolysaccharide-induced macrophages modulate proliferation and invasion of head and neck cancer cell lines. Biomed Pharmacother. 2018;101:988–995. 29635909 10.1016/j.biopha.2018.03.033

[52] Ye X, Wang R, Bhattacharya R, Boulbes DR, Fan F, Xia L, Adoni H, Ajami NJ, Wong MC, Smith DP, et al. Fusobacterium nucleatum subspecies Animalis influences proinflammatory cytokine expression and monocyte activation in human colorectal tumors. Cancer Prev Res (Phila). 2017;10:398–409. 28483840 10.1158/1940-6207.CAPR-16-0178

[53] Sheng D, Yue K, Li H, Zhao L, Zhao G, Jin C, Zhang L. The interaction between Intratumoral microbiome and immunity is related to the prognosis of ovarian cancer. Microbiol Spectr. 2023;11: Article e0354922. 36975828 10.1128/spectrum.03549-22 PMC10100779

[54] Sharma G, Sharma A, Kim I, Cha DG, Kim S, Park ES, Noh JG, Lee J, Ku JH, Choi YH, et al. A dietary commensal microbe enhances antitumor immunity by activating tumor macrophages to sequester iron. Nat Immunol. 2024;25(5):790–801. 38664585 10.1038/s41590-024-01816-x

[55] Zhang W, Xu L, Zhang X, Xu J, Jin JO. Escherichia coli adhesion portion FimH polarizes M2 macrophages to M1 macrophages in tumor microenvironment via toll-like receptor 4. Front Immunol. 2023;14:1213467. 37720226 10.3389/fimmu.2023.1213467 PMC10502728

[56] Fan L, Xu C, Ge Q, Lin Y, Wong CC, Qi Y, Ye B, Lian Q, Zhuo W, Si J, et al. Muciniphila suppresses colorectal tumorigenesis by inducing TLR2/NLRP3-mediated M1-like TAMs. Cancer Immunol Res. 2021;9(10):1111–1124. 34389559 10.1158/2326-6066.CIR-20-1019

[57] Mirji G, Worth A, Bhat SA, El Sayed M, Kannan T, Goldman AR, Tang HY, Liu Q, Auslander N, Dang CV, et al. The microbiome-derived metabolite TMAO drives immune activation and boosts responses to immune checkpoint blockade in pancreatic cancer. Sci Immunol. 2022;7(75): Article eabn0704. 36083892 10.1126/sciimmunol.abn0704 PMC9925043

[58] Wei W, Li J, Shen X, Lyu J, Yan C, Tang B, Ma W, Xie H, Zhao L, Cheng L, et al. Oral microbiota from periodontitis promote oral squamous cell carcinoma development via γδ t cell activation. mSystems. 2022;7(5): Article e0046922. 36000726 10.1128/msystems.00469-22 PMC9600543

[59] Xu C, Fan L, Lin Y, Shen W, Qi Y, Zhang Y, Chen Z, Wang L, Long Y, Hou T, et al. Fusobacterium nucleatum promotes colorectal cancer metastasis through miR-1322/CCL20 axis and M2 polarization. Gut Microbes. 2021;13(1):1980347. 34632963 10.1080/19490976.2021.1980347 PMC8510564

[60] Li R, Zhou R, Wang H, Li W, Pan M., Yao X, Zhan W, Yang S, Xu L, Ding Y, et al. Gut microbiota-stimulated cathepsin K secretion mediates TLR4-dependent M2 macrophage polarization and promotes tumor metastasis in colorectal cancer. Cell Death Differ*.* 2019;26(11):2447–2463. 30850734 10.1038/s41418-019-0312-y PMC6889446

[61] Ma Y, Chen H, Li H, Zheng M, Zuo X, Wang W, Wang S, Lu Y, Wang J, Li Y, et al. Intratumor microbiome-derived butyrate promotes lung cancer metastasis. Cell Rep Med. 2024;5(4): Article 101488. 38565146 10.1016/j.xcrm.2024.101488 PMC11031379

[62] Saito S, Cao DY, Maekawa T, Tsuji NM, Okuno A. Lactococcus lactis subsp. cremoris C60 upregulates macrophage function by modifying metabolic preference in enhanced anti-tumor immunity. Cancers. 2024;16(10):1928. 38792006 10.3390/cancers16101928 PMC11120145

[63] Hwang CH, Kim KT, Lee NK, Paik HD. Immune-enhancing effect of heat-treated Levilactobacillus brevis KU15159 in RAW 264.7 cells. Probiotics Antimicrob Proteins. 2023;15:175–184. 36178579 10.1007/s12602-022-09996-4 PMC9523639

[64] Jung JY, Shin JS, Lee SG, Rhee YK, Cho CW, Hong HD, Lee KT. Lactobacillus sakei K040706 evokes immunostimulatory effects on macrophages through TLR 2-mediated activation. Int Immunopharmacol. 2015;28(1):88–96. 26049027 10.1016/j.intimp.2015.05.037

[65] Feng G, Xu Y, Ma N, Midorikawa K, Oikawa S, Kobayashi H, Nakamura S, Ishinaga H, Zhang Z, Huang G, et al. Influence of Epstein-Barr virus and human papillomavirus infection on macrophage migration inhibitory factor and macrophage polarization in nasopharyngeal carcinoma. BMC Cancer. 2021;21(1):929. 34407796 10.1186/s12885-021-08675-x PMC8371777

[66] Xu X, Zhu N, Zheng J, Peng Y, Zeng MS, Deng K, Duan C, Yuan Y. EBV abortive lytic cycle promotes nasopharyngeal carcinoma progression through recruiting monocytes and regulating their directed differentiation. PLOS Pathog. 2024;20(1): Article e1011934. 38206974 10.1371/journal.ppat.1011934 PMC10846743

[67] Wang J, Luo Y, Bi P, Lu J, Wang F, Liu X, Zhang B, Li X. Mechanisms of Epstein-Barr virus nuclear antigen 1 favor Tregs accumulation in nasopharyngeal carcinoma. Cancer Med. 2020;9(15):5598–5608. 32573058 10.1002/cam4.3213 PMC7402843

[68] Srisathaporn S, Ekalaksananan T, Heawchaiyaphum C, Aromseree S, Maranon DG, Altina NH, Nukpook T, Wilusz J, Pientong C. EBV-induced LINC00944: A driver of oral cancer progression and influencer of macrophage differentiation. Cancers. 2025;17(3):491. 39941858 10.3390/cancers17030491 PMC11815735

[69] Gandhi MK, Hoang T, Law SC, Brosda S, O’Rourke K, Tobin JWD, Vari F, Murigneux V, Fink L, Gunawardana J, et al. EBV-associated primary CNS lymphoma occurring after immunosuppression is a distinct immunobiological entity. Blood. 2021;137(11):1468–1477. 33202420 10.1182/blood.2020008520 PMC7976507

[70] Chen Y, Ouyang D, Wang Y, Pan Q, Zhao J, Chen H, Yang X, Tang Y, Wang Q, Li Y, et al. EBV promotes TCR-T-cell therapy resistance by inducing CD163+M2 macrophage polarization and MMP9 secretion. J Immunother Cancer. 2024;12(6): Article e008375. 38886114 10.1136/jitc-2023-008375 PMC11184188

[71] Seminerio I, Kindt N, Descamps G, Bellier J, Lechien JR, Mat Q, Pottier C, Journé F, Saussez S. High infiltration of CD68+ macrophages is associated with poor prognoses of head and neck squamous cell carcinoma patients and is influenced by human papillomavirus. Oncotarget. 2018;9:11046–11059. 29541395 10.18632/oncotarget.24306 PMC5834277

[72] Kindt N, Descamps G, Lechien JR, Remmelink M, Colet JM, Wattiez R, Berchem G, Journe F, Saussez S. Involvement of HPV infection in the release of macrophage migration inhibitory factor in head and neck squamous cell carcinoma. J Clin Med. 2019;8(1):75. 30634708 10.3390/jcm8010075 PMC6352225

[73] Gilardini Montani MS, Falcinelli L, Santarelli R, Granato M, Romeo MA, Cecere N, Gonnella R, D’Orazi G, Faggioni A, Cirone M. KSHV infection skews macrophage polarisation towards M2-like/TAM and activates Ire1 α-XBP1 axis up-regulating pro-tumorigenic cytokine release and PD-L1 expression. Br J Cancer. 2020;123(2):298–306. 32418990 10.1038/s41416-020-0872-0 PMC7374093

[74] Dai L, Qiao J, Del Valle L, Qin Z. KSHV co-infection regulates HPV16+ cervical cancer cells pathogenesis in vitro and in vivo. Am J Cancer Res. 2018;8(4):708–714. 29736315 PMC5934560

[75] Ou J, Zhu M, Ju X, Xu D, Lu G, Li K, Jiang W, Wan C, Zhao Y, Han Y, et al. One-dimensional rod-like tobacco mosaic virus promotes macrophage polarization for a tumor-suppressive microenvironment. Nano Lett. 2023;23(5):2056–2064. 36695738 10.1021/acs.nanolett.2c03809

[76] Khamaru S, Mukherjee T, Tung KS, Kumar PS, Bandyopadhyay S, Mahish C, Chattopadhyay S, Chattopadhyay S. Chikungunya virus infection inhibits B16 melanoma-induced immunosuppression of T cells and macrophages mediated by interleukin 10. Microb Pathog. 2024;197: Article 107022. 39419458 10.1016/j.micpath.2024.107022

[77] Weinberg JB, Hibbs JB Jr. Enhanced macrophage tumoricidal activity and tumor suppression or regression caused by heat-killed Candida albicans. J Natl Cancer Inst. 1979;63:1273–1278. 388016

[78] Wang X, Wu S, Wu W, Zhang W, Li L, Liu Q, Yan Z. Candida albicans promotes oral cancer via IL-17A/IL-17RA-macrophage axis. mBio. 2023;14: Article e0044723. 37067414 10.1128/mbio.00447-23 PMC10294694

[79] Zhao K, Ma Y, Luo J, Xu Y, Shou Q, Jiang H, Zhu X. Hirsutella sinensis fungus promotes CD8(+) T cell-mediated anti-tumor immunity by affecting tumor-associated macrophages-derived CCRL2. Immunol Investig. 2025;54:573–588. 39819245 10.1080/08820139.2025.2450246

[80] Wang B, Li Q, Wang J, Zhao S, Nashun B, Qin L, Chen X. Plasmodium infection inhibits tumor angiogenesis through effects on tumor-associated macrophages in a murine implanted hepatoma model. Cell Commun Signal. 2020;18(1):157. 32972437 10.1186/s12964-020-00570-5 PMC7513281

[81] Wei Z, Chen G, Zhang P, Zhu L, Zhang L, Chen K. Rhizopus nigricans polysaccharide activated macrophages and suppressed tumor growth in CT26 tumor-bearing mice. Carbohydr Polym. 2018;198:302–312. 30093003 10.1016/j.carbpol.2018.06.076

[82] Liu C, He D, Zhang S, Chen H, Zhao J, Li X, Zeng X. Homogeneous polyporus polysaccharide inhibit bladder cancer by resetting tumor-associated macrophages toward M1 through NF-kappaB/NLRP3 signaling. Front Immunol. 2022;13: Article 839460. 35603205 10.3389/fimmu.2022.839460 PMC9115861

[83] Luo S, Huang X, Li S, Chen Y, Zhang X, Zeng X. Homogeneous Polyporus polysaccharide exerts anti-bladder cancer effects via autophagy induction. Pharm Biol. 2024;62(2):214–221. 38353262 10.1080/13880209.2024.2316195 PMC10868468

[84] Zong S, Li J, Ye Z, Zhang X, Yang L, Chen X, Ye M. Lachnum polysaccharide suppresses S180 sarcoma by boosting anti-tumor immune responses and skewing tumor-associated macrophages toward M1 phenotype. Int J Biol Macromol. 2020;144:1022–1033. 31669462 10.1016/j.ijbiomac.2019.09.179

[85] Jin Y, Fu L. Engineer a double team of short-lived and glucose-sensing bacteria for cancer eradication. Cell Rep Med. 2023;4(6): Article 101043. 37192627 10.1016/j.xcrm.2023.101043 PMC10313919

[86] Wu L, Du Z, Li L, Qiao L, Zhang S, Yin X, Chang X, Li C, Hua Z. Camouflaging attenuated Salmonella by cryo-shocked macrophages for tumor-targeted therapy. Signal Transduct Target Ther. 2024;9(1):14. 38195682 10.1038/s41392-023-01703-1 PMC10776584

[87] Xu H, Piao L, Wu Y, Liu X. IFN-γ enhances the antitumor activity of attenuated salmonella-mediated cancer immunotherapy by increasing M1 macrophage and CD4 and CD8 T cell counts and decreasing neutrophil counts. Front Bioeng Biotechnol. 2022;10: Article 996055. 36246355 10.3389/fbioe.2022.996055 PMC9556780

[88] Wu L, Qiao L, Zhang S, Qiu J, Du Z, Sun Y, Chang X, Li L, Li C, Qiao X, et al. Dual-engineered macrophage-microbe encapsulation for metastasis immunotherapy. Adv Mater. 2024;36(36): Article e2406140. 39023382 10.1002/adma.202406140

[89] Zhou C, Chen J, Zheng B, Zhu P, Chu Q, Li F, Fu Y, Li X, Luo J. Integration of CoAl-layered double hydroxides on commensal bacteria to enable targeted tumor inhibition and immunotherapy. ACS Appl Mater Interfaces. 2023;15(38):44731–44741. 37708438 10.1021/acsami.3c08936

[90] Peters L, Weidenfeld I, Klemm U, Loeschcke A, Weihmann R, Jaeger KE, Drepper T, Ntziachristos V, Stiel AC. Phototrophic purple bacteria as optoacoustic in vivo reporters of macrophage activity. Nat Commun. 2019;10(1):1191. 30867430 10.1038/s41467-019-09081-5 PMC6416252

[91] Wang Y, Fan Y, Zhang X, Liu J, Sun D, Li L, Bai G, Liu X, Kang J, Zhang Y, et al. In situ production and precise release of bioactive GM-CSF and siRNA by engineered bacteria for macrophage reprogramming in cancer immunotherapy. Biomaterials. 2025;317: Article 123037. 39729775 10.1016/j.biomaterials.2024.123037

[92] Rezaei Adriani R, Mousavi Gargari SL, Bakherad H, Amani J. Anti-EGFR bioengineered bacterial outer membrane vesicles as targeted immunotherapy candidate in triple-negative breast tumor murine model. Sci Rep. 2023;13(1):16403. 37775519 10.1038/s41598-023-43762-y PMC10541432

[93] Feng Q, Ma X, Cheng K, Liu G, Li Y, Yue Y, Liang J, Zhang L, Zhang T, Wang X, et al. Engineered bacterial outer membrane vesicles as controllable two-way adaptors to activate macrophage phagocytosis for improved tumor immunotherapy. Adv Mater. 2022;34(40): Article e2206200. 35985666 10.1002/adma.202206200

[94] Fu Z, Lin S, Chen H, Guo H, Li J, Chen Y, Lu Y, Liu J, Huang W, Pang Y. Generating self-adjuvated nanofiber vaccines by coating bacterial flagella with antigens. Adv Mater. 2025;37(13): Article e2415887. 39981905 10.1002/adma.202415887

[95] Xie XT, Guan M, Cheng K, Li Y, Zhang B, Zhou YT, Tan LF, Dong PS, Chen S, Liu B, et al. Programmable engineered bacteria as sustained-releasing antibody factory in situ for enhancing tumor immune checkpoint therapy. Sci Adv. 2025;11(13): Article eadt7298. 40138400 10.1126/sciadv.adt7298 PMC11939038

[96] Xu X, Ding Y, Dong Y, Yuan H, Xia P, Qu C, Ma J, Wang H, Zhang X, Zhao L, et al. Nanobody-engineered biohybrid bacteria targeting gastrointestinal cancers induce robust STING-mediated anti-tumor immunity. Adv Sci. 2024;11(31): Article e2401905. 10.1002/advs.202401905 PMC11336900 38888519

[97] Zhu L, Liu J, Qiu M, Chen J, Liang Q, Peng G, Zou Z. Bacteria-mediated metformin-loaded peptide hydrogel reprograms the tumor immune microenvironment in glioblastoma. Biomaterials. 2022;288: Article 121711. 35948494 10.1016/j.biomaterials.2022.121711

[98] Zhao Z, Chung YH, Steinmetz NF. Melanoma immunotherapy enabled by M2 macrophage targeted immunomodulatory cowpea mosaic virus. Mater Adv. 2024;5(4):1473–1479. 38380336 10.1039/d3ma00820g PMC10876082

[99] Wang C, Steinmetz NF. CD47 blockade and cowpea mosaic virus nanoparticle in situ vaccination triggers phagocytosis and tumor killing. Adv Healthc Mater. 2019;8(8): Article e1801288. 30838815 10.1002/adhm.201801288 PMC6633909

[100] Li M, Zhang Y, Zong L, Zhang M, Wang S, Lei W, Qian W. An oncolytic vaccinia virus expressing anti-CD47 nanobody exerts enhanced antitumor activity by mediating innate and adaptive immune cell infiltration and activation in the lymphoma tumor microenvironment. Haematologica. 2025;110(9):2113–2128. 40207734 10.3324/haematol.2024.286923 PMC12399946

[101] Pol J, Kroemer G, Galluzzi L. First oncolytic virus approved for melanoma immunotherapy. Onco Targets Ther. 2016;5(5): Article e1115641. 10.1080/2162402X.2015.1115641 PMC4760283 26942095

[102] Wang S, Kong L, Wang L, Zhuang Y, Guo C, Zhang Y, Cui H, Gu X, Wu J, Jiang C. Viral expression of NE/PPE enhances anti-colorectal cancer efficacy of oncolytic adenovirus by promoting TAM M1 polarization to reverse insufficient effector memory/effector CD8^+^ T cell infiltration. J Exp Clin Cancer Res. 2025;44(1):97. 40082916 10.1186/s13046-025-03358-y PMC11907943

[103] Huang Y, Lv SQ, Liu PY, Ye ZL, Yang H, Li LF, Zhu HL, Wang Y, Cui LZ, Jiang DQ, et al. A SIRPα-Fc fusion protein enhances the antitumor effect of oncolytic adenovirus against ovarian cancer. Mol Oncol. 2020;14(3):657–668. 31899582 10.1002/1878-0261.12628 PMC7053234

[104] Zhang Y, He B, Zou P, Wu M, Wei M, Xu C, Dong J, Wei J. Targeted release of a bispecific fusion protein SIRPα/Siglec-10 by oncolytic adenovirus reinvigorates tumor-associated macrophages to improve therapeutic outcomes in solid tumors. J Immunother Cancer. 2025;13(4): Article e010767. 40169285 10.1136/jitc-2024-010767 PMC11962785

[105] Governa V, Brittoli A, Mele V, Pinamonti M, Terracciano L, Muenst S, Iezzi G, Spagnoli GC, Zajac P, Trella E. A replication-incompetent CD154/40L recombinant vaccinia virus induces direct and macrophage-mediated antitumor effects in vitro and in vivo. Onco Targets Ther. 2019;8(5): Article e1568162. 10.1080/2162402X.2019.1568162 PMC6492963 31069131

[106] Yan W, Xuan Y, Wang R, Huan Z, Guo Y, Dun H, Xu L, Han R, Sun X, Si L, et al. Oncolytic vaccinia virus armed with GM-CSF and IL-7 enhances antitumor immunity in pancreatic cancer. Biomedicine. 2025;13(4):882. 10.3390/biomedicines13040882 PMC12024586 40299475

[107] Parviainen S, Ahonen M, Diaconu I, Kipar A, Siurala M, Vähä-Koskela M, Kanerva A, Cerullo V, Hemminki A. GMCSF-armed vaccinia virus induces an antitumor immune response. Int J Cancer. 2015;136(5):1065–1072. 25042001 10.1002/ijc.29068

[108] Zhang G, Wang Q, Yuan R, Zhang Y, Chen K, Yu J, Ye T, Jia X, Zhou Y, Li G, et al. Oncolytic vaccinia virus harboring aphrocallistes vastus lectin exerts anti-tumor effects by directly oncolysis and inducing immune response through enhancing ROS in human ovarian cancer. Biochem Biophys Res Commun. 2024;730: Article 150355. 38996784 10.1016/j.bbrc.2024.150355

[109] Yao Y, Li B, Chen C, Wang J, Yao F, Li Z. HVEM as a tumor-intrinsic regulator in non-small cell lung cancer: Suppression of metastasis via glycolysis inhibition and modulation of macrophage polarization. Pharmacol Res. 2025;213: Article 107604. 39832683 10.1016/j.phrs.2025.107604

[110] Jackson JW, Hall BL, Marzulli M, Shah VK, Bailey L, Chiocca EA, Goins WF, Kohanbash G, Cohen JB, Glorioso JC. Treatment of glioblastoma with current oHSV variants reveals differences in efficacy and immune cell recruitment. Mol Ther Oncolytics. 2021;22:444–453. 34553031 10.1016/j.omto.2021.07.009 PMC8430372

[111] Zhu L, Huang J, Zhang S, Cai Q, Guo X, Liu B, Chen L, Zheng C. oHSV2-mGM repolarizes TAMs and cooperates with αPD1 to reprogram the immune microenvironment of residual cancer after radiofrequency ablation. Biomed Pharmacother. 2024;178: Article 117060. 39053421 10.1016/j.biopha.2024.117060

[112] Sun T, Wang M, Zhang L, Gong M, Xie Q, Yang X, Xiao S, Zhang W, Liu X, Zhao Y, et al. Engineered bacterial biohybrid-mediated CD47-SIRPα blockade and HSP90 inhibition for enhanced immuno-photothermal therapy. ACS Appl Mater Interfaces. 2025;17(20):29183–29197. 40331355 10.1021/acsami.5c01645

[113] Zhao J, Huang H, Zhao J, Xiong X, Zheng S, Wei X, Zhou S. A hybrid bacterium with tumor-associated macrophage polarization for enhanced photothermal-immunotherapy. Acta Pharm Sin B. 2022;12:2683–2694. 35755281 10.1016/j.apsb.2021.10.019 PMC9214064

[114] Wen AM, Lee KL, Cao P, Pangilinan K, Carpenter BL, Lam P, Veliz FA, Ghiladi RA, Advincula RC, Steinmetz NF. Utilizing viral nanoparticle/dendron hybrid conjugates in photodynamic therapy for dual delivery to macrophages and cancer cells. Bioconjug Chem. 2016;27(5):1227–1235. 27077475 10.1021/acs.bioconjchem.6b00075 PMC5160013

[115] Chen K, Li H, Xu Y, Ge H, Ning X. Photoactive “bionic virus” robustly elicits the synergy anticancer activity of immunophotodynamic therapy. ACS Appl Mater Interfaces. 2022;14(3):4456–4468. 35021012 10.1021/acsami.1c23983

[116] Ye LY, Li YS, Ge T, Liu LC, Si JX, Yang X, Fan WJ, Liu XZ, Zhang YN, Wang JW, et al. Engineered luminescent oncolytic vaccinia virus activation of photodynamic-immune combination therapy for colorectal cancer. Adv Healthc Mater. 2024;13(17): Article e2304136. 38551143 10.1002/adhm.202304136

[117] Xie S, Zhang P, Zhang Z, Liu Y, Chen M, Li S, Li X. Bacterial navigation for tumor targeting and photothermally-triggered bacterial ghost transformation for spatiotemporal drug release. Acta Biomater. 2021;131:172–184. 34171461 10.1016/j.actbio.2021.06.030

[118] Meng J, Li L, Xu Y, Wei X, Chen D, Chen C, Liu S, Wang Z, Shi G, Wang S, et al. A simple liposome-based bionic bacterium for tumor treatment by re-education of tumor-associated microphages in combination with chemotherapy. Colloids Surf B Biointerfaces. 2023;222: Article 113069. 36508889 10.1016/j.colsurfb.2022.113069

[119] Davar D, Carneiro BA, Dy GK, Sheth S, Borad MJ, Harrington KJ, Patel SP, Galanis E, Samson A, Agrawal S, et al. Phase I study of a recombinant attenuated oncolytic virus, MEDI5395 (NDV-GM-CSF), administered systemically in combination with durvalumab in patients with advanced solid tumors. J Immunother Cancer. 2024;12(11): Article e009336. 39551600 10.1136/jitc-2024-009336 PMC11574399

[120] Yang Y, Wang Y, Zeng F, Chen Y, Chen Z, Yan F. Ultrasound-visible engineered bacteria for tumor chemo-immunotherapy. Cell Rep Med. 2024;5(5): Article 101512. 38640931 10.1016/j.xcrm.2024.101512 PMC11148858

[121] Pei P, Zhang Y, Jiang Y, Shen W, Chen H, Yang S, Zhang Y, Yi X, Yang K. Pleiotropic immunomodulatory functions of radioactive inactivated bacterial vectors for enhanced cancer radio-immunotherapy. ACS Nano. 2022;16(7):11325–11337. 35819107 10.1021/acsnano.2c04982

[122] Chen WY, Chen YL, Lin HW, Chang CF, Huang BS, Sun WZ, Cheng WF. Stereotactic body radiation combined with oncolytic vaccinia virus induces potent anti-tumor effect by triggering tumor cell necroptosis and DAMPs. Cancer Lett. 2021;523:149–161. 34606928 10.1016/j.canlet.2021.09.040

[123] Zhang Y, Tan W, Sultonova RD, Nguyen DH, Zheng JH, You SH, Rhee JH, Kim SY, Khim K, Hong Y, et al. Synergistic cancer immunotherapy utilizing programmed Salmonella typhimurium secreting heterologous flagellin B conjugated to interleukin-15 proteins. Biomaterials. 2023;298: Article 122135. 37148758 10.1016/j.biomaterials.2023.122135

[124] Wu L, Li L, Li S, Liu L, Xin W, Li C, Yin X, Xu X, Bao F, Hua Z. Macrophage-mediated tumor-targeted delivery of engineered Salmonella typhi murium VNP20009 in anti-PD1 therapy against melanoma. Acta Pharm Sin B. 2022;12(10):3952–3971. 36213533 10.1016/j.apsb.2022.05.006 PMC9532557

[125] Li Y, Wang X, Ye F, Hong X, Chen Y, Huang J, Liu J, Huang X, Liang L, Guo Y, et al. Acid-responsive engineered bacteria with aberrant in-situ anti-PD-1 expression for post-ablation immunotherapy of hepatocellular carcinoma. Biomed Pharmacother. 2025;186: Article 118046. 40209305 10.1016/j.biopha.2025.118046

[126] Meng F, Li L, Zhang Z, Lin Z, Zhang J, Song X, Xue T, Xing C, Liang X, Zhang X. Biosynthetic neoantigen displayed on bacteria derived vesicles elicit systemic antitumour immunity. J Extracell Vesicles. 2022;11(12); Article e12289. 36468941 10.1002/jev2.12289 PMC9721206

[127] Guo F, Das JK, Kobayashi KS, Qin QM, Ficht TA, Alaniz RC, Song J, Figueiredo P. Live attenuated bacterium limits cancer resistance to CAR-T therapy by remodeling the tumor microenvironment. J Immunother Cancer. 2022;10(1): Article e003760. 34987022 10.1136/jitc-2021-003760 PMC8734016

[128] An JX, Han ZY, Qin YT, Li CX, He JH, Zhang XZ. Bacteria-based backpacks to enhance adoptive macrophage transfer against solid tumors. Adv Mater. 2024;36(6): Article e2305384. 37672674 10.1002/adma.202305384

[129] Xuan Y, Yan W, Wang R, Wang X, Guo Y, Dun H, Huan Z, Xu L, Han R, Sun X, et al. GM-CSF and IL-21-armed oncolytic vaccinia virus significantly enhances anti-tumor activity and synergizes with anti-PD1 immunotherapy in pancreatic cancer. Front Immunol. 2024;15:1506632. 39830516 10.3389/fimmu.2024.1506632 PMC11739091

[130] Gu J, Xu X, Li X, Yue L, Zhu X, Chen Q, Gao J, Takashi M, Zhao W, Zhao B, et al. Correction: Tumor-resident microbiota contributes to colorectal cancer liver metastasis by lactylation and immune modulation. Oncogene. 2025;44(24):2004–2007. 40456867 10.1038/s41388-025-03439-4 PMC12143973

[131] Epstein AL, Rabkin SD. Safety of non-replicative and oncolytic replication-selective HSV vectors. Trends Mol Med. 2024;30(8):781–794. 38886138 10.1016/j.molmed.2024.05.014 PMC11329358

[132] Sachin K, Karn SK. Microbial fabricated nanosystems: Applications in drug delivery and targeting. Front Chem. 2021;9: Article 617353. 33959586 10.3389/fchem.2021.617353 PMC8093762

[133] Walsh NC, Kenney LL, Jangalwe S, Aryee KE, Greiner DL, Brehm MA, Shultz LD. Humanized mouse models of clinical disease. Annu Rev Pathol. 2017;12(1):187–215. 27959627 10.1146/annurev-pathol-052016-100332 PMC5280554

[134] Yang R, Wang S, Li Z, Yin C, Huang W, Huang W. Patient-derived organoid co-culture systems as next-generation models for bladder cancer stem cell research. Cancer Lett. 2025;625: Article 217793. 40368172 10.1016/j.canlet.2025.217793

[135] Li Y, Cui Z, Song X, Chen Y, Li C, Shi J, Qian W, Ren G, Zhou J, Li C, et al. Single-cell transcriptomic landscape deciphers intratumoral heterogeneity and subtypes of acral and mucosal melanomas. Clin Cancer Res. 2025;31(12):2495–2514. 40192737 10.1158/1078-0432.CCR-24-3164 PMC12163602

[136] Arango-Argoty G, Bikiel DE, Sun GJ, Kipkogei E, Smith KM, Carrasco Pro S, Choe EY, Jacob E. AI-driven predictive biomarker discovery with contrastive learning to improve clinical trial outcomes. Cancer Cell. 43 (2025) 875–890.e878. 40250446 10.1016/j.ccell.2025.03.029

[137] Aponte M, Murru N, Shoukat M. Therapeutic, prophylactic, and functional use of probiotics: A current perspective. Front Microbiol. 2020;11: Article 562048. 33042069 10.3389/fmicb.2020.562048 PMC7516994

[138] Cao M, Deng Y, Hao Q, Yan H, Wang QL, Dong C, Wu J, He Y, Huang LB, Xia X, et al. Single-cell transcriptomic analysis reveals gut microbiota-immunotherapy synergy through modulating tumor microenvironment. Signal Transduct Target Ther. 2025;10(1):140. 40312419 10.1038/s41392-025-02226-7 PMC12045981

[139] Lin A, Jiang A, Huang L, Li Y, Zhang C, Zhu L, Mou W, Liu Z, Zhang J, Cheng Q, et al. From chaos to order: Optimizing fecal microbiota transplantation for enhanced immune checkpoint inhibitors efficacy. Gut Microbes. 2025;17(1):2452277. 39826104 10.1080/19490976.2025.2452277 PMC12716052

